# A CEP215–HSET complex links centrosomes with spindle poles and drives centrosome clustering in cancer

**DOI:** 10.1038/ncomms11005

**Published:** 2016-03-18

**Authors:** Pavithra L. Chavali, Gayathri Chandrasekaran, Alexis R. Barr, Péter Tátrai, Chris Taylor, Evaggelia K. Papachristou, C. Geoffrey Woods, Sreenivas Chavali, Fanni Gergely

**Affiliations:** 1 Cancer Research UK Cambridge Institute, Li Ka Shing Centre, University of Cambridge, Robinson Way, Cambridge CB2 0RE, UK; 2 Department of Medical Genetics, Cambridge Institute for Medical Research, University of Cambridge, Hills Road, Cambridge CB2 0XY, UK; 3 MRC Laboratory of Molecular Biology, Francis Crick Avenue, Cambridge CB2 0QH, UK

## Abstract

Numerical centrosome aberrations underlie certain developmental abnormalities and may promote cancer. A cell maintains normal centrosome numbers by coupling centrosome duplication with segregation, which is achieved through sustained association of each centrosome with a mitotic spindle pole. Although the microcephaly- and primordial dwarfism-linked centrosomal protein CEP215 has been implicated in this process, the molecular mechanism responsible remains unclear. Here, using proteomic profiling, we identify the minus end-directed microtubule motor protein HSET as a direct binding partner of CEP215. Targeted deletion of the HSET-binding domain of CEP215 in vertebrate cells causes centrosome detachment and results in HSET depletion at centrosomes, a phenotype also observed in CEP215-deficient patient-derived cells. Moreover, in cancer cells with centrosome amplification, the CEP215–HSET complex promotes the clustering of extra centrosomes into pseudo-bipolar spindles, thereby ensuring viable cell division. Therefore, stabilization of the centrosome–spindle pole interface by the CEP215–HSET complex could promote survival of cancer cells containing supernumerary centrosomes.

## Abstract (web summary)

Centrosome clustering allows survival of cells with amplified centrosomes at the cost of chromosome instability. Here, Chavali *et al*. show that the centrosome component CEP215 collaborates with the kinesin motor HSET both to maintain spindle poles connections and to cluster centrosomes.

## Graphical abstract

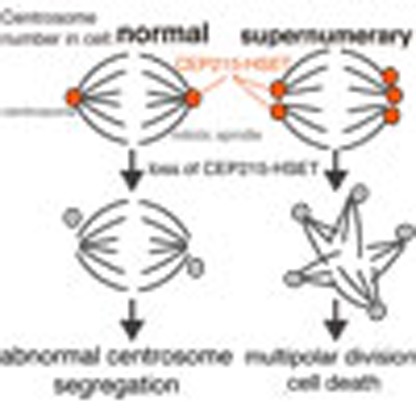

Centrosomes act as dominant sites of microtubule assembly in mitosis and therefore centrosome number corresponds to the number of spindle poles formed^1^. Because faithful transmission of genetic information requires a bipolar mitotic spindle, centrosome numbers must be tightly controlled in cells. Accordingly, centrosome numbers are regulated by two mechanisms. First, centrosome duplication is limited to once per cell cycle ensuring that cells enter mitosis with two functional centrosomes^2^,^3^. Second, each centrosome associates and co-segregates with its own mitotic spindle pole causing each daughter cell to inherit precisely one centrosome^4^. Centrosomes and mitotic spindle poles are distinct structures, well illustrated by the presence of focused spindle poles in cells lacking centrosomes^5^,^6^,^7^. Spindle pole formation relies on microtubule motors and microtubule-associated proteins that crosslink and focus bundles of kinetochore-associated microtubules (k-fibres).

In *Drosophila* S2 cells the key protein responsible for holding centrosomes at spindle poles is dynein, a minus end-directed motor^8^,^9^,^10^. Dynactin increases the processivity of dynein and together they transport the spindle pole integrity protein, nuclear mitotic apparatus (NuMA) to the minus ends of spindle microtubules^11^,^12^. In NuMA-deficient mammalian cells, k-fibres lose focus and centrosomes detach from the poles^13^. Similar phenotypes have been documented in *Drosophila* cells and embryos upon disruption of the minus end-directed kinesin-14 motor protein, non-claret-disjunctional (ncd)^10^,^14^. By contrast, the mammalian homologue HSET is largely dispensable for k-fibre focus. Instead, HSET contributes to spindle elongation through crosslinking and sliding microtubules, functions dependent on its C-terminal motor domain and the additional microtubule-binding site in its N-terminal tail^15^. Both ncd and HSET have been implicated in survival of cells with centrosome amplification^16^,^17^,^18^,^19^. In particular, the orthologues mediate clustering of supernumerary centrosomes into pseudo-bipolar spindles, a role essential for continued proliferation of cells with centrosome amplification. HSET also promotes clustering of acentrosomal spindle poles^17^.

The centrosome comprises a pair of centrioles embedded in the pericentriolar matrix (PCM), the site of microtubule nucleation. CEP215 is an evolutionarily conserved PCM protein present in microtubule-organizing centres from yeast to human; the centrosomin motif 1 (CM1) in its N terminus binds the γ-tubulin complex^20^,^21^,^22^,^23^. CEP215 organizes several PCM components including pericentrin and AKAP450 (refs 24, 25, 26, 27, 28, 29, 30). Deletion of centrosomin (cnn), its *Drosophila* orthologue, disruption of the CM1 domain of chicken CEP215 and depletion of CEP215 in HeLa cells all cause centrosome detachment from mitotic spindle poles^27^,^31^,^32^. However, spindle pole focus is maintained in CM1-deficient cells, consistent with normal localization of NuMA and dynactin^27^. Mutations in CEP215 are associated with congenital diseases such as primary microcephaly and primordial dwarfism^33^,^34^.

Here we set out to identify the molecular mechanism by which CEP215 maintains centrosome attachment to spindle poles. We identify HSET as a direct interactor of CEP215 and demonstrate that HSET binding by CEP215 is crucial for its role in this process. We further show that cancer cells with centrosome amplification rely on the CEP215–HSET complex for centrosome clustering and survival.

## Results

### Identification of CEP215-interacting partners in DT40 cells

**Figure 1:**
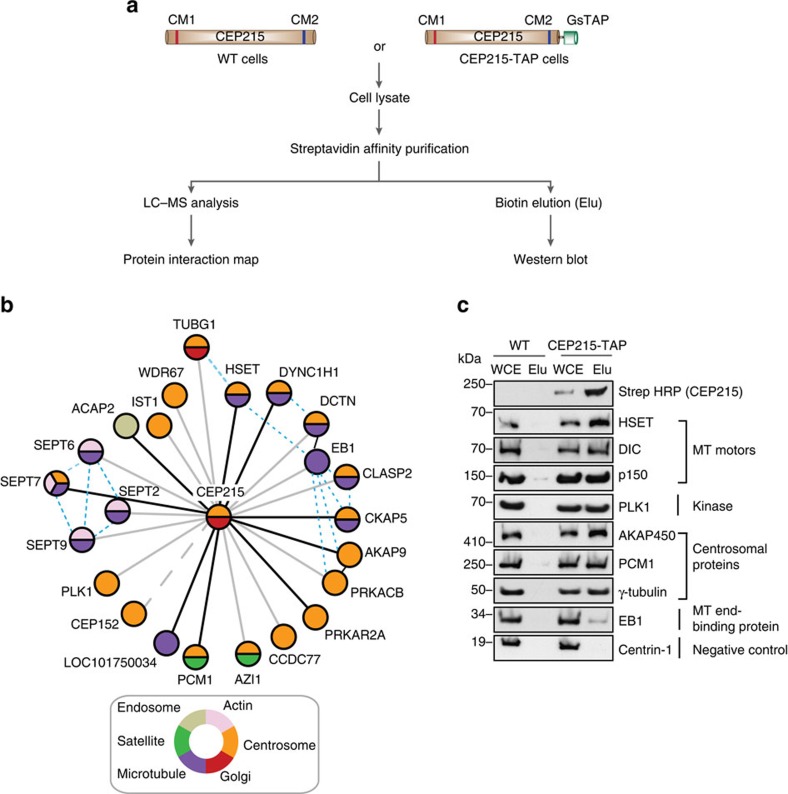
Protein interaction network of CEP215. (**a**) Schematic representation of the workflow used to identify interacting partners of CEP215. (**b**) The interactome map was constructed based on the mass spectrometric analysis of affinity-purified TAP-CEP215-containing protein complexes. GsTAP tag consists of protein G and streptavidin-binding protein. Each node represents a binding partner of CEP215 identified in all three biological replicates, but absent in WT cells and detected by a minimum of four unique peptides in at least two replicates (Supplementary Table 1). Actual or predicted subcellular localization of proteins are colour coded. The greater a Mascot score (best of three replicates), the darker the corresponding line. Dashed line for CEP152 refers to protein being found only in two experiments. Blue dashed lines mark previously reported binding between interactors of CEP215. (**c**) Whole-cell extracts (WCE) of WT or TAP-CEP215 cells were subjected to affinity purification (Elu) and immunoblotted with the indicated antibodies. DIC, dynein intermediate chain; MT, microtubule.

To establish the molecular basis for CEP215 function in centrosome–spindle pole attachment, we employed an unbiased proteomic approach to isolate and identify CEP215 interactors. To this end, affinity purification tags (GsTAP containing protein G and streptavidin-binding protein) were inserted in-frame into both alleles of the CEP215 gene (CEP215-TAP cell line) in the chicken B cell line, DT40 (refs 27, 35). Following affinity purification, protein complexes were analysed by mass spectrometry (Fig. 1a; Supplementary Fig. 1). Proteins were considered as hits if they were represented by one or more unique peptides in all three biological replicates and by four or more unique peptides in at least two replicates. We filtered out putative hits if they were represented even by a single unique peptide in pulldowns performed from wild-type (WT) cells. Hits were further filtered against other GsTAP affinity purification experiments to exclude TAP tag-specific binding^36^. An interacting network of CEP215 was constructed based on these criteria (Fig. 1b). All previously reported interacting partners have been identified, in addition to new ones that include PCM1, CKAP5/ch-Tog and HSET, a minus end-directed microtubule motor (Fig. 1b; Supplementary Table 1; Supplementary Data 1). Western blot analysis confirmed interactions (Fig. 1c). Because of its roles in mitotic spindle pole organization in *Drosophila* and cancer cells, we have decided to focus on HSET for the purpose of this study.

### CEP215 and HSET bind directly in vertebrates

**Figure 2:**
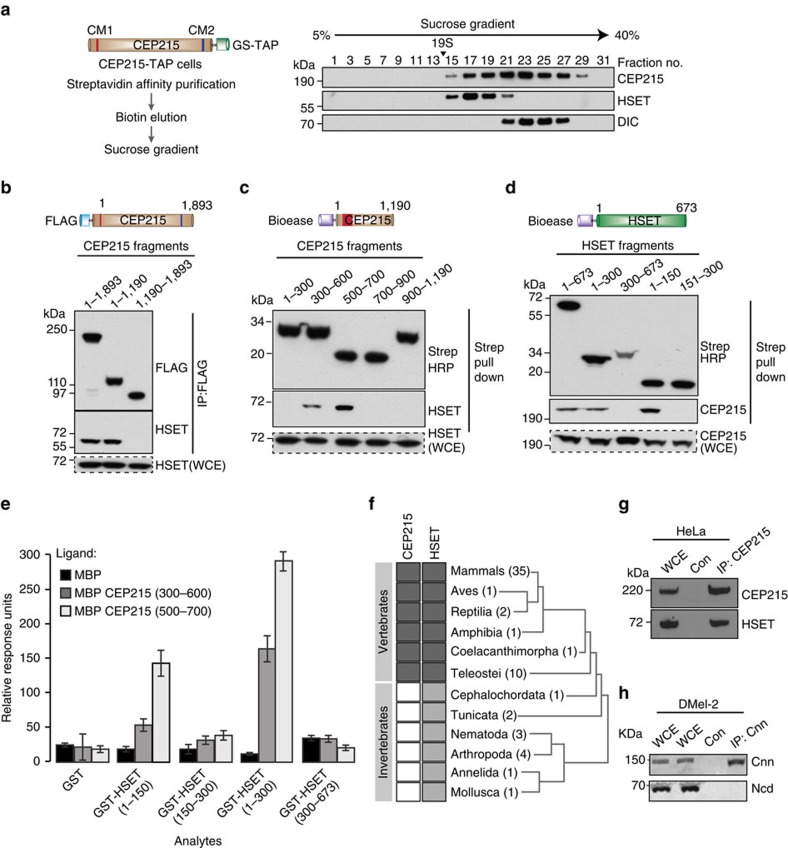
CEP215 and HSET interact through vertebrate-specific binding domains. (**a**) Left panel depicts the workflow for separation of TAP-CEP215-bound complexes on a 5–40% sucrose gradient. Western blots of sucrose fractions probed with antibodies as indicated. (**b**) Whole-cell extracts (WCE) of HeLa cells expressing FLAG-tagged CEP215 fragments were subjected to FLAG pull-down followed by western blotting with the indicated antibodies. (**c**) WCE of CEP215-depleted HeLa cells expressing Bioease-tagged CEP215 fragments as indicated were subjected to streptavidin (strep) pull-down followed by western blotting with the indicated antibodies. (**d**) WCE of HeLa cells expressing Bioease-tagged HSET fragments were subjected to streptavidin (strep) pull-down followed by western blotting with the indicated antibodies. (**e**) HSET and CEP215 bind directly. Graph depicts qualitative analysis of binding between MBP-tagged CEP215 fragments (substrates) and GST-tagged HSET fragments (ligands) using surface plasmon resonance plotted as relative response units. GST and MBP proteins were used as controls. MBP shows background response for each analyte. Values for three technical replicates are shown. Error bars correspond to standard deviation. (**f**) Sequences of HBR of CEP215 and aa1–150 of HSET have been analysed across 97 organisms (Supplementary Fig. 2d). Dark grey cells indicate high sequence conservation within HBR of CEP215 and aa1–150 of HSET. Light grey cells depict lesser conservation of aa1–150 of HSET. Compared with human HSET aa1–150, invertebrates showed an average sequence identity of 12% in contrast to 54% among vertebrates. White cells depict the absence of HBR in CEP215 orthologues. Numbers in parentheses represent the number of organisms per class for which CEP215 and/or HSET sequences are available. (**g**) WCE of mitotic HeLa cells were subjected to immunoprecipitation by an anti-CEP215 antibody or random IgG (con) followed by western blotting with the indicated antibodies. (**h**) WCE of Drosophila Dmel2 cells were subjected to immunoprecipitation by an anti-centrosomin (Cnn) antibody followed by western blotting with the indicated antibodies.

CEP215 interacts with the microtubule motor dynein and its adaptor, dynactin^37^. To establish if HSET, dynein and CEP215 exist in the same complex, CEP215-TAP-containing protein complexes were fractionated on a sucrose gradient. CEP215-bound HSET sedimented at a lower sucrose concentration than CEP215-bound dynein, indicative of separate complexes (Fig. 2a). Gel filtration experiment yielded similar results (Supplementary Fig. 2a).

To further characterize the CEP215-HSET interaction, we elucidated the respective binding domains in human CEP215 and HSET. CEP215 fusion products were expressed in HeLa cells constitutively depleted of CEP215 (Supplementary Fig. 2b). The HSET-binding region was mapped to the two overlapping regions in the N terminus of CEP215: amino acids (aa) 500–700 and 300–600 (Fig. 2b,c). In HSET it is aa1–150 at the N terminus (that is, the tail domain) that binds CEP215 (Fig. 2d). The CEP215–HSET interaction is direct, as suggested by yeast two-hybrid assays and surface plasmon resonance (SPR) (Fig. 2e; Supplementary Fig. 2c). In SPR aa500–700 of CEP215 displayed an ∼2.5-fold greater binding to HSET when compared with aa300–600. We therefore consider aa500–700 of CEP215 as the minimal HSET-binding region (HBR). Sequence analysis of HBR of human CEP215 revealed three helical regions that are conserved in vertebrates. Remarkably, the tail of HSET also shows a high degree of conservation in the vertebrate lineage, raising the possibility that the interaction between HSET and CEP215 arose in this lineage (Fig. 2f; Supplementary Figs 2d, 3 and 4). Indeed, we could not detect binding between *Drosophila* cnn and ncd, the respective homologues of human CEP215 and HSET, whereas the two proteins co-immunoprecipitated in human HeLa cells (Fig. 2g,h). The ancestral cnn gene underwent a duplication event in cephalochordates producing CEP215 and another CM1-containing gene, myomegalin. Unlike CEP215, myomegalin lacks an HBR and, accordingly, failed to interact with HSET (Supplementary Fig. 2e).

### CEP215-HSET complex connects centrosomes to spindle poles

**Figure 3:**
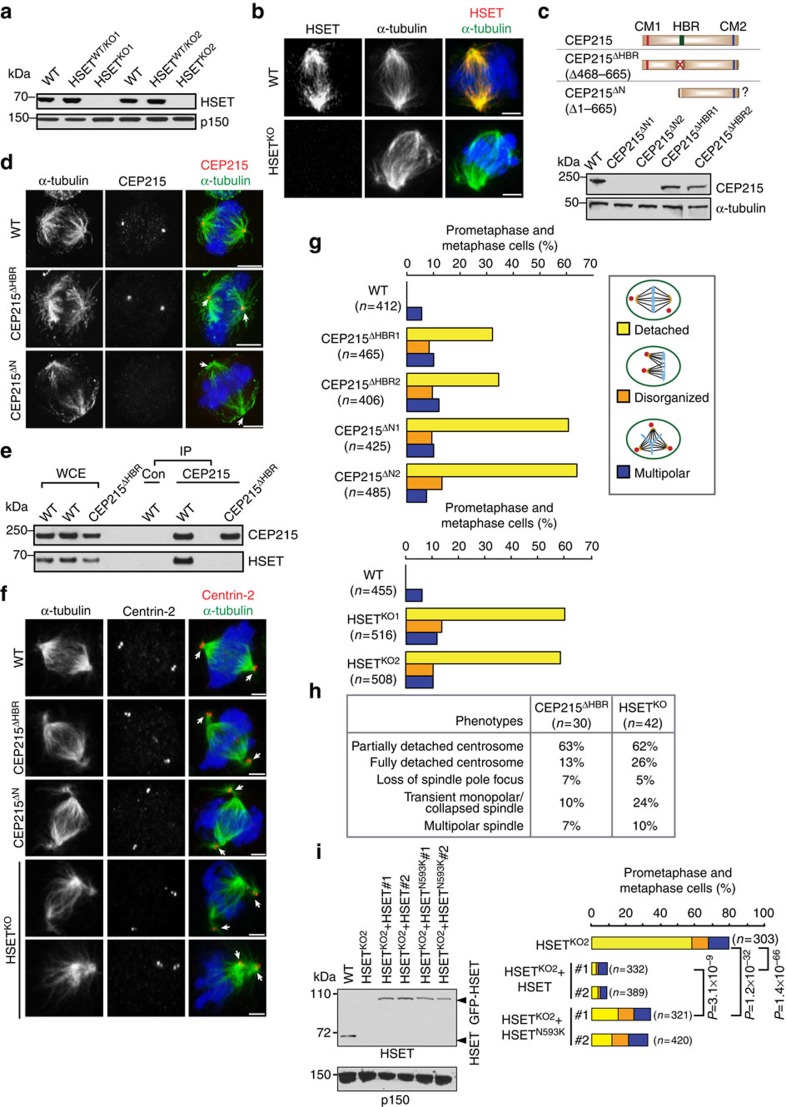
HSET binding by CEP215 is required for association between centrosomes and spindle poles. (**a**) Whole-cell extracts (WCE) of wild-type (WT) DT40, heterozygous and homozygous clones of HSET^KO^ are immunoblotted with an anti-HSET antibody recognizing aa300–673. (**b**) Immunofluorescence images show WT and HSET^KO1^ cells stained for HSET (red) and α-tubulin (green). DNA is in blue. Scale bar, 3 μm. (**c**) Schematics of expected truncations are shown on top. Note that an N-terminally truncated product may be expressed in CEP215^ΔN^. At the bottom, WCE of WT and homozygous clones of CEP215^ΔHBR^ and CEP215^ΔN^ are immunoblotted with an N-terminal anti-CEP215 antibody. (**d**) Representative images show WT, CEP215^ΔHBR^ and CEP215^ΔN^ cells stained for CEP215 (red) and α-tubulin (green). DNA is in blue. Scale bar, 4 μm (**e**) WCE of WT and CEP215 ^ΔHBR^ cells were subjected to immunoprecipitation (IP) by an anti-CEP215 antibody or random IgG (con) followed by western blotting. Antibodies for immunoblotting are indicated. CEP215^ΔHBR^ does not interact with HSET. (**f**) Representative images illustrate mitotic phenotypes in CEP215^ΔN^, CEP215^ΔHBR^ and HSET^KO^ cells stained for centrin-2 (red) and α-tubulin (green). DNA is in blue. Arrows indicate completely or partially detached centrosomes. Bottom panel shows collapsed spindle in HSET^KO^. Scale bar, 4 μm. (**g**) Graph depicts quantification of phenotypes as percentage of total mitotic cells in two independent clones of CEP215^ΔHBR^, CEP215^ΔN^ and HSET^KO^ cells (>500 mitotic cells per clone). (**h**) Table summarizes mitotic phenotypes of CEP215^ΔN^ and HSET^KO^ cells from time-lapse experiments. (**i**) WCE from HSET^KO2^ cells stably transfected with GFP-tagged wild-type HSET (HSET^KO2^-HSET) or mutant HSET^N593K^ (HSET^KO2^-HSET^N593K^) were subjected to western blotting with the indicated antibodies. Graph on right depicts quantification of phenotypes as percentage of total mitotic cells (colours as in *g*). *P* values were obtained by Fisher's exact test. In the graph *P* values are shown for the detachment phenotype. *P* values for the second clones: HSET^KO^ versus HSET^KO^+HSET#2: *P*=1.02 × 10^−69^; HSET^KO^ versus HSET^KO^+HSET(N593K)#2: *P*=1.03 × 10^−43^; HSET^KO^+HSET#2 versus HSET^KO^+HSET(N593K)#2: 2.77 × 10^−6^. *P* values for disorganized spindle are shown in the text.

We next wanted to address the functional significance of the CEP215–HSET interaction. Using gene targeting we created chicken DT40 cell lines in which either HSET or the HBR of CEP215 was disrupted. The HSET knockout line (HSET^KO^) was generated by replacing the exons encoding the tail and stalk domains (aa1–345) with antibiotic resistance genes^38^ (Supplementary Fig. 5a). Using western blots and immunofluorescence, we confirmed that HSET^KO^ cells were protein null (Fig. 3a,b).

The HBR in chicken CEP215 maps to aa482–663. The CEP215^ΔHBR^ cell line was generated through an in-frame fusion of exons 11 and 17, resulting in deletion of aa468–665 (Supplementary Fig. 5b). Since the genomic sequence encoding for HBR spans 12.8 kb, we performed sequential targeting: first, exons 13–16 were removed followed by exon 12. Antibiotic resistance genes were excised using cre recombinase after each round (Supplementary Fig. 5b). As expected, CEP215^ΔHBR^ cells expressed a truncated CEP215 mRNA in which exons 11 and 17 are fused (Supplementary Fig. 5c,d). The corresponding protein product (termed CEP215(ΔHBR)) showed similar expression levels and localization to the wild-type protein, suggestive of normal folding, yet did not interact with HSET (Fig. 3c–e). In addition to CEP215^ΔHBR^, an intermediate cell line called CEP215^ΔN^ was included in our study. In this case exons 13–16 were replaced by antibiotic resistance genes, but these were not excised by cre recombinase (Supplementary Fig. 5b). An antibody against aa40–375 of CEP215 revealed no product in CEP215^ΔN^ cells (Fig. 3c). Thus, even if a truncated protein is produced from the mutant alleles, this product lacks both the CM1 (aa83–141) and HBR domains. mRNA analysis of CEP215^ΔN^ showed a truncated transcript with low expression levels (Supplementary Fig. 5d). All three lines were viable, but exhibited a mild proliferation defect and an elevated mitotic index (Supplementary Fig. 6a,b).

Centrosome detachment was observed in HSET^KO^, CEP215^ΔN^ and CEP215^ΔHBR^ cells (Fig. 3f,g). The category ‘detached' includes cells with one or two partially or completely detached centrosomes. Over 30% of CEP215^ΔHBR^ mitotic cells displayed centrosome detachment, suggesting that HSET binding by CEP215 is vital to maintain centrosomes at spindle poles in DT40 cells. The centrosome detachment phenotype reached ∼60% in HSET^KO^ and CEP215^ΔN^ cells. A further 10% of the mutants displayed multipolar spindles with an additional ∼5–10% of cells showing abnormal spindle morphology ranging from unfocussed spindle to monopolar/collapsed spindles in HSET^KO^ (Fig. 3g). To better understand these phenotypes, mitosis was followed live using GFP-EB3 in HSET^KO^ and CEP215^ΔHBR^ cells (Fig. 3h; Supplementary Movies 1–7). Partial and/or complete centrosome detachment was seen in both HSET^KO^ and CEP215^ΔHBR^. Furthermore, 24% of HSET^KO^ cells showed a transient collapse of the spindle into a monopole soon after nuclear envelope breakdown, revealing a role for HSET in maintenance of bipolarity at the early stages of spindle assembly (Fig. 3h). Nevertheless, all HSET^KO^ cells subsequently regained bipolarity and initiated normal anaphase. Importantly, we found no evidence for loss of centrosome integrity in the mutants: normal PCM organization was confirmed by confocal and 3D-structured illumination microscopy both in spindle pole-associated and detached centrosomes (Supplementary Fig. 6c–e). Consistently, microtubule-nucleating capacity of isolated centrosomes was preserved when tested in *Xenopus* egg extracts (Supplementary Fig. 6f).

Ncd/HSET contains separate microtubule-binding and motor domains that permit microtubule crosslinking and sliding, respectively^39^,^40^,^41^. To address which function is responsible for linking centrosomes with spindle poles, we made use of the N593K point mutation in HSET, which markedly decreases the ATPase and sliding activities of the motor without impacting on its crosslinking function^15^. HSET^KO^ cells were transfected with GFP fusions of wild-type or N593K-mutant human HSET. Single clones (called HSET^KO^-HSET and HSET^KO^-HSET^N593K^) were selected with transgene expression levels comparable to endogenous HSET. GFP-HSET almost fully rescued centrosome detachment and disorganized spindles in HSET^KO^ cells (Fig. 3i). By contrast, GFP-HSET(N593K) reduced centrosome detachment to ∼15%, a significant, but nonetheless inferior rescue when compared with GFP-HSET. Therefore, microtubule crosslinking appears to be the more dominant role of HSET in attaching centrosomes to spindle poles, but sliding also plays a part. Interestingly, GFP-HSET(N593K) was unable to prevent formation of disorganized spindles, suggesting that the motor activity is crucial for HSET function in spindle organization (*P* values for disorganized spindle phenotype: HSET^KO^ versus HSET^KO^+HSET#1: 1.07 × 10^−7^; HSET^KO^ versus HSET^KO^+HSET(N593K): 0.7821486; Fisher's exact tests).

### CEP215 is responsible for centrosomal accumulation of HSET

**Figure 4:**
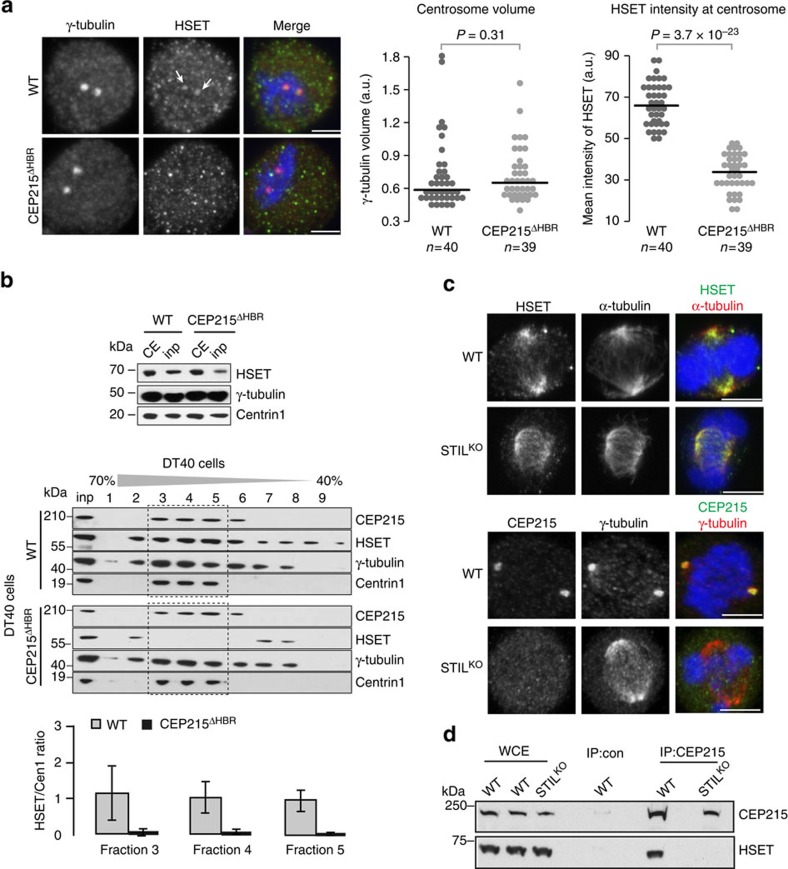
CEP215 promotes association of HSET with centrosomes. (**a**) Images show WT and CEP215^ΔHBR^ cells in which microtubules were depolymerized by nocodazole. Cells are stained for HSET (green) and γ-tubulin (red). Dot plots on right depict the volume of centrosomes (that is, measured as the volume of γ-tubulin-positive structures) and the mean signal intensity of HSET in centrosomes. Note that each dot represents a cell; centrosome volumes and mean HSET intensities were averaged across the two centrosomes in each cell (WT: *n*=40; CEP215^ΔHBR^: *n*=39). *P* values are obtained by Fisher's test. Scale bar, 3 μm. (**b**) Representative western blots of centrosomes isolated from WT and CEP215^ΔHBR^ cells. Western blot on top shows cell lysates before and after centrifugation onto a 50% sucrose cushion to enrich for centrosomes (CE and inp, respectively). This input (inp) was further centrifuged through a discontinuous sucrose gradient (% sucrose is indicated above blots) with results shown on western blots below. Frame depicts centrin-rich fractions corresponding to centrosomes. Antibodies for immunoblotting are indicated. Note reduction of HSET in CEP215^ΔHBR^ centrosomes. Graph below shows quantification of the HSET to centrin-1 signal ratio in centrin-rich fractions; *n*=3 biological replicates. Error bars correspond to standard deviation. (**c**) WT and STIL^KO^ cells in top panels are stained for HSET (green) and α-tubulin (red), and in bottom panels for CEP215 (green) and γ-tubulin (red). DNA is in blue. Scale bar, 4 μm. (**d**) WCE of WT and STIL^KO^ cells were subjected to immunoprecipitation (IP) by random IgG (con) or anti-CEP215 antibody, followed by western blotting using indicated antibodies.

We next asked whether CEP215 could influence localization of HSET to the spindle or centrosomes. HSET localized normally to spindles of CEP215^ΔHBR^ cells (Supplementary Fig. 6g). To measure the centrosomal pool of HSET specifically, microtubules were depolymerized with nocodazole in WT and CEP215^ΔHBR^ DT40 cells (Fig. 4a). HSET signal intensity was then quantified in mitotic centrosomes as defined by the volume of γ-tubulin staining. While centrosome volumes were similar between WT and CEP215^ΔHBR^, HSET levels were significantly reduced at centrosomes (Fig. 4a). Likewise, when centrosomes were isolated by sucrose sedimentation from WT and CEP215^ΔHBR^ cells, a marked decrease in HSET was seen in the latter (Fig. 4b). These findings raised the possibility that the CEP215–HSET interaction might occur at centrosomes. We tested the idea using the STIL^KO^ DT40 cell line that lacks functional centrosomes^7^. In STIL^KO^ cells HSET is present, whereas CEP215 is absent from the spindle apparatus (Fig. 4c)^7^. Strikingly, immunoprecipitation of CEP215 in STIL^KO^ cells revealed loss of interaction with HSET, implying that intact centrosomes are a prerequisite of CEP215–HSET complex formation (Fig. 4d). We conclude that CEP215 is likely to bind HSET at centrosomes, which in turn increases centrosomal levels of HSET.

### HBR and CM1 domains of CEP215 scaffold distinct interactions

**Figure 5:**
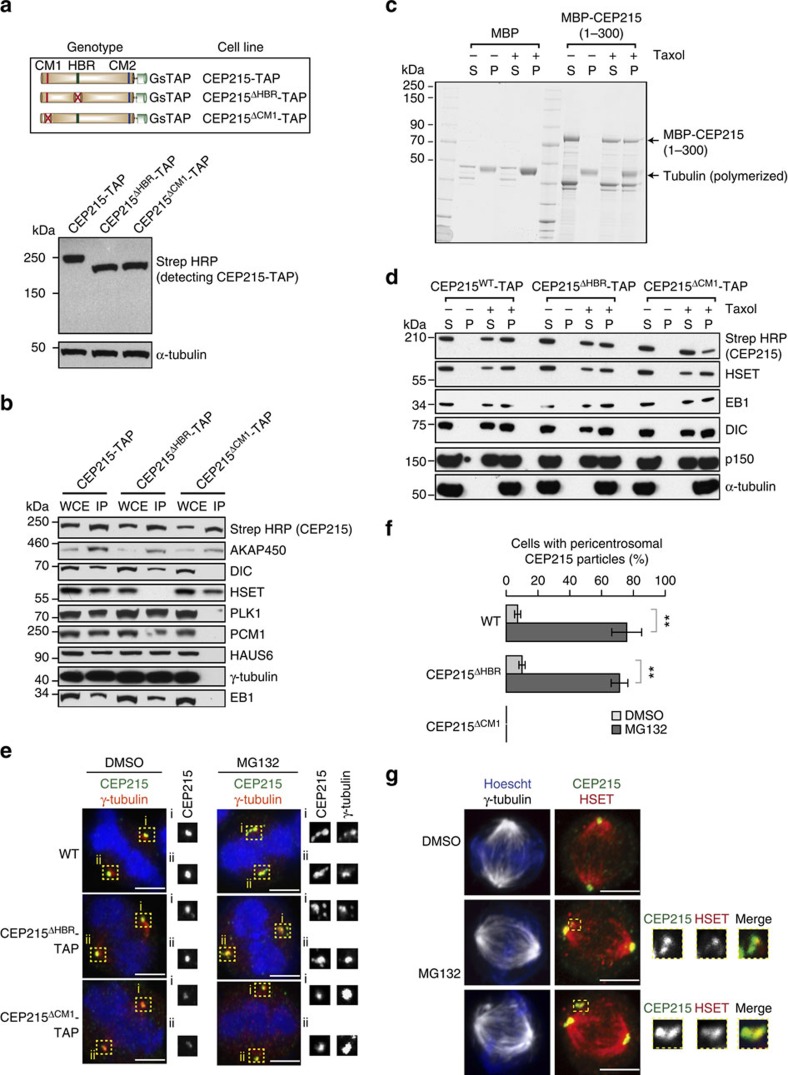
HBR of CEP215 mediates HSET binding exclusively, whereas its CM1 domain is responsible for multiple interactions. (**a**) Table depicts summary of TAP-tagged cell lines. The panel below shows the expression of protein products from CEP215-TAP, CEP215^ΔHBR^-TAP and CEP215^ΔCM1^-TAP cell lines. (**b**) CEP215-containing protein complexes were affinity purified from CEP215-TAP, CEP215^ΔHBR^-TAP and CEP215^ΔCM1^-TAP cells, followed by western blotting for indicated antibodies. (**c**) Binding of MBP-CEP215 (1–300) to microtubules was assayed using microtubule spin-down in the presence of tubulin (−taxol) or taxol-stabilized (+taxol) microtubules. MBP served as a negative control. Following centrifugation supernatants (S) and pellets (P) were loaded on gel and stained with Coomassie blue. (**d**) Microtubule spin-down assays were performed from lysates of CEP215-TAP, CEP215^ΔHBR^-TAP and CEP215^ΔCM1^-TAP cells in the presence of tubulin (−taxol) or taxol-stabilized microtubules (+taxol). Following centrifugation supernatants (S) and pellets (P) were subjected to western blotting. Antibodies for immunoblotting are indicated. Arrowhead marks the panel depicting the reduction of CEP215(ΔCM1)-TAP binding to microtubules. (**e**) Pericentrosomal CEP215 particles are visualized in DMSO- and MG132-treated WT, CEP215^ΔHBR^ and CEP215^ΔCM1^ cells. Cells were stained for CEP215 (green) and γ-tubulin (red). DNA is in blue. Arrow highlights a particle. Insets show higher magnification of CEP215 and γ-tubulin stainings corresponding to framed areas. Scale bar, 4 μm. (**f**) Graphs show quantitation of pericentrosomal CEP215 particles as percentage of mitotic cells in the presence of DMSO or MG132. *P* values of paired *t*-tests (*P<0.05, **P<0.005); *n*=3 biological replicates. Error bars correspond to standard deviation. (**g**) DMSO- and MG132-treated WT cells were stained for CEP215 (green) and HSET (red). DNA is in blue. Scale bar, 4 μm. Insets show higher magnification of CEP215 and HSET stainings corresponding to framed areas. Note co-localization of the two proteins on pericentrosomal particles.

Our group previously reported centrosome detachment in a cell line where the first 140 aa of CEP215, containing the centrosomin motif 1 (CM1), were deleted (called CEP215^ΔCM1^)^27^. Because disruption of CM1 decreases centrosomal levels of CEP215 by nearly 70%, the observed centrosome detachment phenotype (∼50%) could reflect the combined effect of CM1 deletion and reduced centrosomal accumulation of CEP215. These findings have nonetheless raised the question of how the CM1 and HBR domains contribute to the function of CEP215 at the centrosome–spindle pole interface. To address this point, CEP215^ΔCM1^-TAP and CEP215^ΔHBR^-TAP cells were generated through biallelic insertion of GsTAP tags into the respective mutant CEP215 loci (Fig. 5a). As in Fig. 1, we employed TAP affinity purification to uncover binding partners of the truncated proteins. Remarkably, except for HSET, CEP215(ΔHBR)-TAP precipitated every interactor from Fig. 1c. By contrast, CEP215(ΔCM1)-TAP could bind HSET, but failed to precipitate γ-tubulin, dynein, PCM1 and Plk1 kinase amongst others (Fig. 5b).

Sequences within CM1 have been shown to activate γ-tubulin complexes *in vitro*, albeit this interaction does not seem relevant to the mitotic role of CEP215 (refs 21, 22, 30). Therefore, we wondered if this highly conserved domain could also bind microtubules. Bacterially expressed aa1–300 of CEP215 co-pelleted with microtubules, indicative of direct binding (Fig. 5c). Moreover, microtubule spin-down experiments from cell lysates revealed a 3.4-fold reduction in microtubule binding of CEP215(ΔCM1)-TAP when compared with CEP215-TAP and CEP215(ΔHBR)-TAP (Fig. 5d). Collectively, our data demonstrate that CEP215 utilizes HBR exclusively for HSET binding, whereas the CM1 domain mediates microtubule association and a host of other interactions.

### CEP215 and HSET co-localize on pericentrosomal particles

We showed that binding between CEP215 and HSET requires intact centrosomes (Fig. 4d). However, the CEP215–HSET complex was isolated from affinity purification experiments performed on cytoplasmic lysates, and not on centrosomal fractions, indicating that some of the complex is associated only loosely with centrosomes and/or may even leave the organelle. In fly embryos GFP-fused Cnn/CEP215 appear on centrosome ‘flares', PCM particles that detach from centrosomes^42^. We therefore wondered if similar structures existed in vertebrate cells, and if so, whether these contained HSET. Flare-like CEP215 staining was detected in ∼8% of WT mitotic DT40 cells (Fig. 5e,f). Treatment with the proteasome inhibitor MG132 raised centrosomal CEP215 levels and concomitantly increased the percentage of cells with pericentrosomal CEP215 particles to over 70% both in WT and CEP215^ΔHBR^ cells (Fig. 5e,f). As in flies, these particles decreased upon depolymerization of microtubules by nocodazole (Supplementary Fig. 7a)^42^. HSET was visible in these structures, suggesting that CEP215–HSET may travel on these pericentrosomal particles in a microtubule-dependent fashion (Fig. 5g). Interestingly, such particles were absent in CEP215^ΔCM1^ cells, although this could be due to lower levels of CEP215(ΔCM1) at centrosomes both in DMSO- and MG132-treated cells (Fig. 5e)^27^.

Pericentriolar satellites are small granules that surround the centrosome in interphase and are thought to disperse during mitosis^43^. Since the core satellite component, PCM1, was present in the CEP215 interaction network (Fig. 1b), we tested if flares in mitotic DT40 cells could correspond to satellites. However, this is unlikely to be the case, since we found no evidence for PCM1 enrichment in the flares (Supplementary Fig. 7b).

### Reduced HSET in centrosomes of CEP215 mutant patient cells

**Figure 6:**
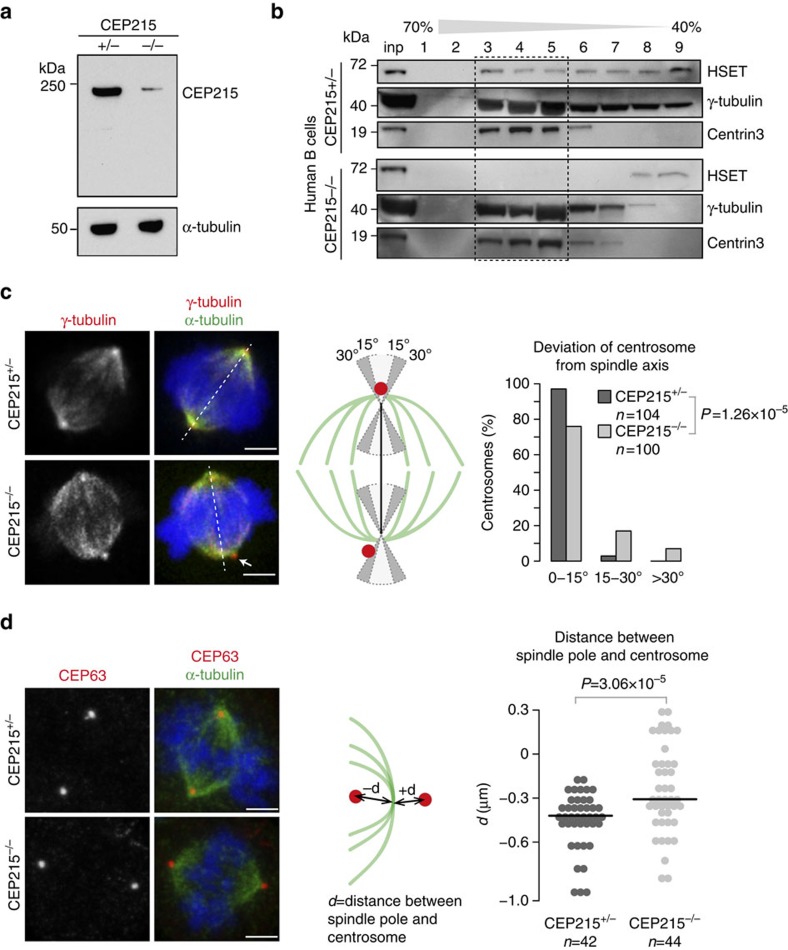
Centrosomes from CEP215 mutant patient cells contain reduced levels of HSET and show mild displacement from spindle poles. (**a**) Whole-cell extracts were prepared from CEP215^+/−^ and CEP215^−/−^ human B lymphocytes followed by western blotting with the indicated antibodies. CEP215 was detected by an antibody against aa900–950. (**b**) Representative western blots of centrosomes isolated from CEP215^+/−^ and CEP215^−/−^ human B lymphocytes. Cell lysates were enriched for centrosomes by centrifugation onto a 50% sucrose cushion (inp) followed by centrifugation through a discontinuous sucrose gradient (% sucrose is indicated above blots). Antibodies for immunoblotting are indicated. (**c**) CEP215^+/−^ and CEP215^−/−^ human lymphocytes were sequentially stained for α-tubulin (green) and γ-tubulin (red). Spindle axis (marked as white dotted line) was determined using automated image analysis (see Methods for details). Position of centrosomes with respect to the axis was determined manually as depicted in schematics and data is shown in a bar chart. Arrow points to a centrosome positioned over 30° from spindle axis. *P* values were obtained by Fisher's exact test for *n*=100 cells. Scale bar, 3 μm. (**d**) Images show CEP215^+/−^ and CEP215^−/−^human lymphocytes stained for the centrosomal protein CEP63 (red) and α-tubulin (green). Dot plot depicts distribution of distance between centrosomes and corresponding spindle poles (CEP215^+/−^: *n*=42 and CEP215^−/−^: *n*=44 cells). *P* values are obtained by Wilcoxon-rank sum test. Scale bar=3 μm.

Mutations in CEP215 cause autosomal recessive primary microcephaly^33^. We have derived parent-of-patient and patient B lymphocytes (CEP215^+/−^ and CEP215^−/−^, respectively) that carry the premature stop codon 243 T>A (S81X) in exon 4 of *CDK5RAP2/CEP215* (ref. 33). On western blots of CEP215^−/−^ cells an antibody against the C terminus of CEP215 revealed a 78% reduction in the intensity of a band similar in size to full-length CEP215 (Fig. 6a). As in chicken cells, centrosomes isolated from patient-derived CEP215^−/−^ B cells contained less HSET than their CEP215^+/−^ counterparts (Fig. 6b).

Although only 2% of CEP215^−/−^ lymphocytes showed centrosome detachment, 24% exhibited centrosomes that appeared at an angle greater than 15° with respect to the spindle axis (3% in CEP215^+/−^; Fig. 6c). We also measured the distance between centrosomes and spindle poles and found it increased in CEP215^−/−^ cells (Fig. 6d). Moreover, we noted that whereas centrosomes were contained within the spindle pole in almost all CEP215^+/−^ cells, they seemed to be outside the spindle poles in nearly 25% of CEP215^−/−^, indicating an outward displacement in the mutants.

Depletion of HSET or CEP215 in HeLa cells also produced centrosome displacement phenotypes, but none replicated the complete centrosome detachment seen in DT40 cells^15^,^37^ (Supplementary Fig. 8a). Several not mutually exclusive explanations exist for the milder phenotype seen in human cells. First, residual CEP215 might be sufficient to maintain centrosome attachment to spindle poles. Second, there may be a partially redundant pathway to CEP215–HSET in human cells, such as that mediated by spindle pole component WDR62, which has no obvious orthologues in chicken^44^. Third, forces—external or internal to the spindle—could contribute to the phenotype and these may vary between species and cell types^45^,^46^. The ratio of centrosomal microtubules versus k-fibres could influence internal forces; this may be skewed in DT40 cells, which have a diploid chromosome number of 78 (normal genome size in chicken), accompanied by weak astral microtubules in mitosis. In addition, external forces could also vary due to differences in cortical organization and cell adhesion^45^. We found that depolymerization of actin in HSET^KO^ and CEP215^ΔHBR^ cells by cytochalasin D reduced the incidence of centrosome detachment in both mutants (Supplementary Fig. 8b). Thus, actomyosin contributes to the centrosome detachment phenotype, probably by increasing forces on the centrosome–spindle pole interface.

### CEP215–HSET promotes centrosome clustering in cancer cells

Cells with centrosome amplification must cluster their supernumerary centrosomes into a pseudo-bipolar spindle for survival, and HSET plays a vital role in this process^18^,^47^. Since our study has identified a functional interaction between HSET and CEP215 in centrosome–spindle pole attachment, we reasoned that CEP215 could also be involved in centrosome clustering. We have therefore examined loss-of-function phenotypes of CEP215 in two cell lines with centrosome amplification: the mouse neuroblastoma line N1E-115 and the human breast cancer cell line BT459, with respective incidences of >99% and ∼25% supernumerary centrosomes. Both BT459 and N1E-115 cells depend on HSET for survival^17^,^18^.

**Figure 7:**
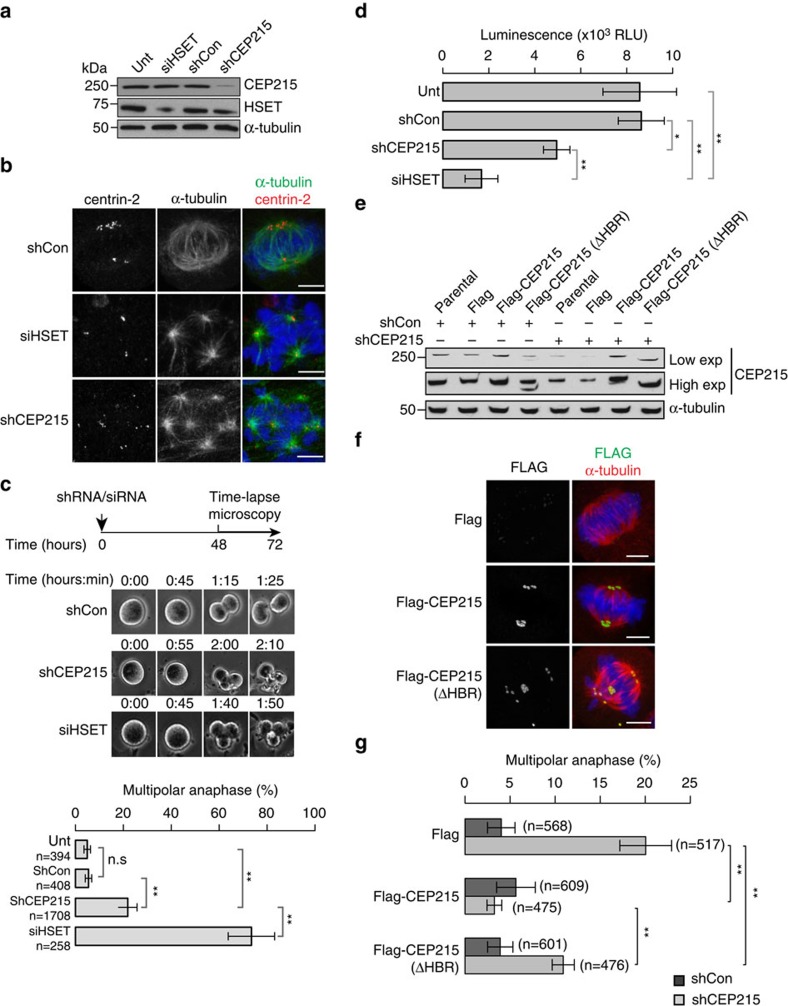
CEP215 facilitates centrosome clustering in mouse neuroblastoma cells via its HBR domain. (**a**) Western blots of whole-cell extracts of N1E-115 cells untreated (unt) or transfected with HSET siRNA (siHSET), retroviral control shRNA (shCon) or CEP215 shRNA (shCEP215) with the indicated antibodies. (**b**) Images show siRNA/shRNA-treated N1E-115 cells stained for centrin-2 (red) and α-tubulin (green)) Scale bar, 8 μm. (**c**) Experimental timeline is shown in schematic. Still frames from time-lapse experiments depict mitosis in untreated (unt) or siRNA/shRNA-treated N1E-115 cells. Graph below shows percentage of mitotic cells with multipolar anaphases from time-lapse experiments. Total number of mitoses analysed per treatment is shown. Two-way ANOVA followed by Tukey's test were performed (**P<0.005); *n*=3 biological replicates. Error bars correspond to standard deviation. (**d**) Graph depicts viability of untreated (unt) or siRNA/shRNA-treated N1E-115 cells as a function of relative light units (RLU) using CellTiter-Glo assay. *P* values of paired *t*-tests (**P<0.005); *n*=3 biological replicates. (**e**) N1E-115 cells stably expressing Flag, Flag-CEP215 or Flag-CEP215(ΔHBR) were transduced with a control (shCon) or CEP215 shRNA (shCEP215) and 72 h later immunoblotted for indicated antibodies. Both low and high exposures of the blot are presented. (**f**) Images show N1E-115 cells stably expressing Flag, Flag-CEP215 or Flag-CEP215(ΔHBR) stained for FLAG (green) and α-tubulin (red). DNA is in blue. Scale bar, 8 μm. (**g**) Parental N1E-115 cells or those stably expressing Flag, Flag-CEP215 or Flag-CEP215(ΔHBR) were transduced with control (shCon) or CEP215 shRNA (shCEP215) and followed live with same timeline as in panel *c*. Graph shows the percentage of mitotic cells with multipolar anaphases from time-lapse experiments. Total number of mitoses analysed per treatment is shown. Two-way ANOVA followed by Tukey's test were performed (**P<0.005); *n*=4 biological replicates. Error bars correspond to s.d.

Because small interfering (si) RNAs were ineffective, we used retroviral small hairpin (sh) RNA to deplete CEP215 in N1E-115 cells, achieving 64% depletion after 72 h (Fig. 7a; Supplementary Fig. 9a). In both CEP215- and HSET-depleted cells we noted an increase in multipolar spindles along with a range of aberrant spindle conformations (Fig. 7b). Multipolar anaphases in live cells were used as a measure of inefficient centrosome clustering. Nearly all N1E-115 cells exhibit bipolar anaphases after resolving multipolar spindle intermediates into pseudo-bipolar spindles. In line with previous reports, time-lapse analysis of N1E-115 siRNA-mediated depletion of HSET caused multipolar anaphases in ∼70% of cells^18^,^48^, whereas 22% of CEP215-depleted cells displayed multipolar anaphases (Fig. 7c; Supplementary Movies 8 and 9). Consistently, cell survival was reduced in both cases (Fig. 7d).

We next asked if HSET binding by CEP215 contributed to its function in centrosome clustering. To this end, we generated single clones of N1E-115 cells by stably expressing Flag only or Flag fusions of human CEP215 or CEP215(ΔHBR). These clones were then transduced with control shRNAs or shRNAs specific to mouse CEP215 (Fig. 7e; Supplementary Fig. 9b). While both FLAG fusion products localized to centrosomes, Flag-CEP215(ΔHBR) exhibited reduced efficacy in centrosome clustering (Fig. 7f). Because Flag-CEP215(ΔHBR) can still mediate some clustering, other sequences in CEP215 such as CM1 might also contribute to CEP215 function in this process (Fig. 7g).

**Figure 8:**
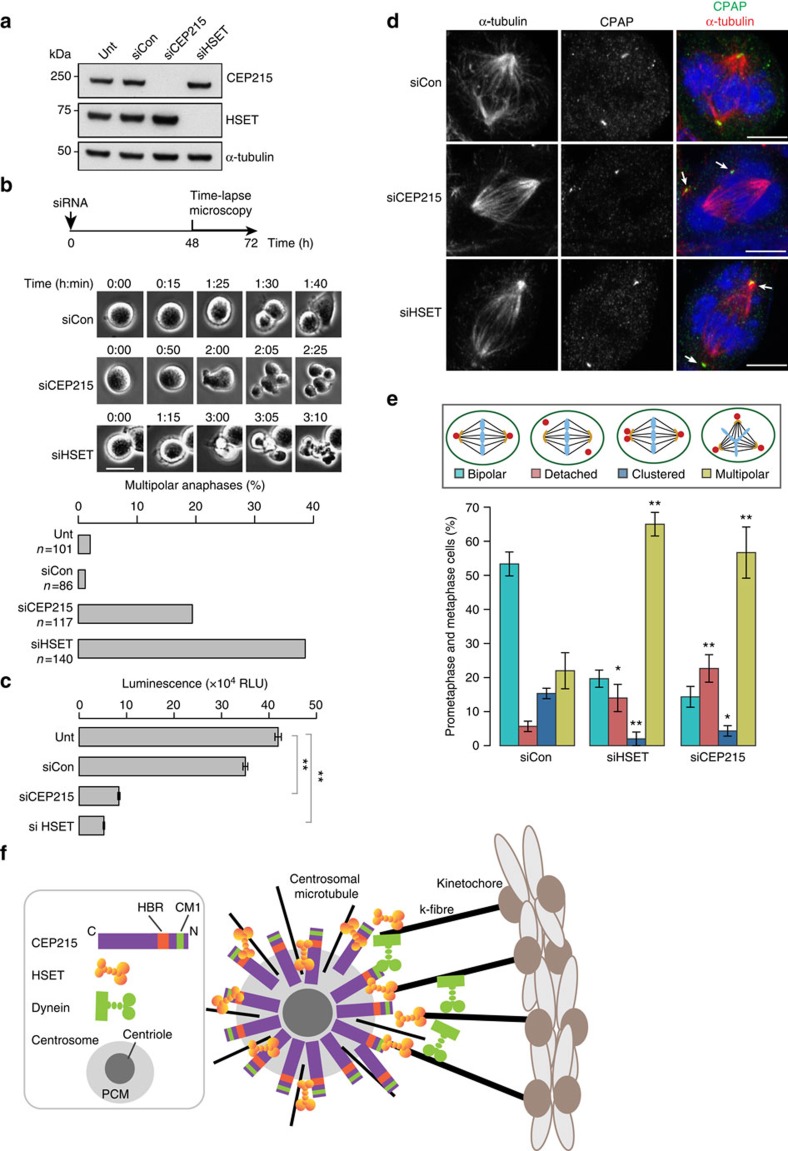
CEP215 and HSET promote centrosome association with mitotic spindle poles and centrosome clustering in human breast cancer cells. (**a**) Western blots of whole-cell extracts of BT-549 cells prepared 72 h after siRNA transfections. Untreated (unt) cells are included as controls. Antibodies for immunoblotting are indicated. (**b**) Still frames from a time-lapse experiment show mitosis in BT-549 cells untreated (unt) or treated with control (siCon), CEP215 (siCEP215) or HSET (siHSET) siRNAs. Graph depicts the number of multipolar anaphases in cells treated with the indicated siRNAs. Total number of mitoses analysed per treatment is shown. (**c**) Graph shows viability of untreated (unt) or siRNA-treated BT-549 cells as a function of relative light units (RLU) using CellTiter-Glo assay. *n*=3 biological replicates, where error bars denote standard deviation and statistical significance was computed using paired *t*-test. (**d**) Images of siRNA-treated BT-549 cells stained for the centriolar marker CPAP (green) and α-tubulin (red). DNA is in blue. Arrows mark detached centrosomes. Scale bar, 6 μm. (**e**) Graphs show quantifications of centrosome and spindle phenotypes (as depicted in schematics) in siRNA-treated BT-549 cells. Two-way ANOVA followed by Tukey's test were performed (*P<0.05, **P<0.005); *n*=3 biological replicates. Error bars correspond to standard deviation. (**f**) Schematic representation of the proposed function of CEP215 at the centrosome–spindle pole interface. Briefly, through HBR CEP215 captures HSET- bound minus ends of k-fibres and interpolar microtubules, thereby anchoring these at the centrosome. CEP215 may also capture dynein-associated microtubules through the CM1 domain.

In BT-549 breast cancer cells a 94% depletion of CEP215 levels was achieved by siRNA (Fig. 8a). Cells were analysed with immunofluorescence and time-lapse microscopy. Both revealed an increase in multipolar spindles as well as multipolar anaphases upon CEP215 knockdown with a concomitant reduction in cell survival (Fig. 8b,c). While analysing centrosome clustering, we noted centrosome detachment in BT-549 cells (Fig. 8d). Detachment was seen in cells with bipolar and multipolar spindles. However, due to the prevalence of acentrosomal spindle poles in these cells^17^, we scored centrosome detachment only in cells that contained a bipolar spindle and two centrosomes. As in DT40 cells, depletion of CEP215 and HSET both triggered centrosome detachment (Fig. 8e).

## Discussion

Centrosomes and spindle poles harbour distinct microtubule populations: the former contains predominantly astral microtubules, whereas the latter contains k-fibres and interpolar microtubules^49^,^50^. Therefore, centrosomes and spindle poles experience different forces, calling for an active mechanism to link the two structures during mitosis^4^. Here we describe a vertebrate-specific interaction between CEP215 and the motor protein HSET, which is required for connecting centrosomes with mitotic spindle poles. Formation of the CEP215–HSET complex requires intact centrosomes and CEP215 promotes centrosomal accumulation of HSET.

Our current understanding of how centrosomes and spindle poles are connected stems from experiments in *Drosophila* S2 cells, where dynein plays a central role by transporting microtubules as well as crosslinking k-fibres with astral microtubules^10^,^51^,^52^. In vertebrate cells removal of astral microtubules does not trigger centrosome detachment, and instead centrosomes move closer to spindle poles, suggesting a nonessential role for astral microtubules in maintaining centrosomes at spindle poles (Supplementary Fig. 10). In mammalian cells centrosome detachment has been observed upon loss of spindle pole focus (that is, disruption of NuMA^13^) or following depletion of the spindle pole protein WDR62 or the centromere component CENP-32, although in these cases the molecular mechanisms are still unclear^44^,^53^,^54^. Intriguingly, CENP-32 depletion leads to a reduction in CEP215 and AKAP450 at mitotic centrosomes^53^. Moreover, like *CEP215*, mutations in *WDR62* cause microcephaly, indicating that an impaired spindle pole–centrosome interface could preclude normal brain development^55^,^56^.

What could be the molecular mechanism by which the CEP215–HSET complex holds centrosomes at spindle poles? We propose a model whereby CEP215 through its HBR captures HSET-bound microtubules, resulting in centrosomal anchoring of k-fibres and interpolar microtubules by CEP215–HSET (Fig. 8f). NuMA and dynein have been shown to accumulate on free microtubule minus ends and facilitate the processive poleward movement of these microtubules^57^. Interestingly, our mass spectrometry analysis of CEP215-binding partners has identified not only dynein but also NuMA, albeit the latter was present in only two experiments. Therefore, CEP215 may also contribute to capturing dynein/NuMA-bound microtubule ends, perhaps through the CM1 domain. This could explain why centrosome detachment is less frequent in CEP215^ΔHBR^ cells than in CEP215^ΔN^ cells where both CM1 and HBR domains are missing. Within the centrosome CEP215 appears to be positioned with its N terminus pointing towards the cytoplasm; such configuration is ideal for the CM1 and HBR domains to capture motors and incoming microtubules^28^.

Impaired centrosome–spindle pole attachment can cause abnormal centrosome segregation, which can lead to supernumerary centrosomes. Indeed, HSET and CEP215 knockout cells displayed an increase in spindle multipolarity (Fig. 3g–i). A hypomorphic mouse model of CEP215 also exhibits centrosome amplification and multipolar spindles in the developing brain, phenotypes observed upon *in utero* siRNA-mediated depletion of CEP215 as well^29^,^58^. Likewise, CEP215-deficient mouse embryonic fibroblasts contain extra centrosomes^26^.

Centrosome clustering in cancer cells with centrosome amplification relies on a range of processes that include the spindle assembly checkpoint, matrix adhesion, microtubule minus end motors dynein and HSET, the chromosome passenger complex and various microtubule-associated proteins^18^,^59^,^60^,^61^. Microtubule attachment and spindle tension seem a prerequisite for efficient clustering^59^. Since centrosome clustering also requires cortical actomyosin forces that act on astral microtubules, these forces must be transmitted from the spindle pole to the centrosome and vice versa^18^. By stabilizing the centrosome–spindle pole connection, CEP215–HSET may coincidentally increase the efficiency of centrosome clustering. In fact, multipolar spindle arrangements could pose the ultimate challenge for centrosome and spindle pole connection. In these unbalanced and asymmetric spindle configurations k-fibre numbers, spindle forces and geometries can differ from pole to pole, as can centrosome size and microtubule nucleation capacity.

In N1E-115 and BT-549 cells depletion of HSET triggers a more severe declustering phenotype than that observed upon CEP215 knockdown. Moreover, ncd/HSET is required for centrosome clustering in flies and also for focusing acentrosomal spindle poles in flies and mammals^15^,^41^,^60^. In these cases the complex is probably irrelevant, because CEP215 and ncd do not seem to interact in flies and require centrosomes to interact in vertebrates. These findings indicate that HSET has CEP215-independent functions in centrosome clustering that are likely to involve sliding and crosslinking of parallel microtubules.

Nonetheless, an interesting conclusion of our study is the vertebrate lineage-specific interaction between CEP215 and HSET, raising the question as to why vertebrate cells have acquired new complexes to secure the connection between centrosomes and spindle poles. With larger genome sizes chromosome numbers often increase, leading to an increase in k-fibre numbers and possibly greater forces at the centrosome–spindle pole interface. Furthermore, whereas in *Drosophila* centrosomes are nonessential for development beyond the syncytial stages, loss of centrosomes causes embryonic lethality in mice, and absence of centrosomes triggers p53-dependent apoptosis in cultured mammalian somatic cells^6^,^62^,^63^,^64^. By facilitating a stable association of centrosomes with spindle poles and thereby correct centrosome segregation, the CEP215–HSET complex could promote cell survival in vertebrates; if so, CEP215 deficiency is expected to cause cell loss, consistent with the primordial dwarfism phenotype seen in patients with mutations in CEP215 (ref. 34).

## Methods

### Cell culture and drug treatments

DT40 and human B cells were cultured as described previously^27^,^36^. BT-549 cells were cultured in RPMI medium supplemented with 10% fetal bovine serum (FBS) and 0.023 IU insulin. HeLa and ecotropic Phoenix cells were cultured in DMEM medium with 10% FBS. HeLa cells were obtained from Jonathan Pines (Gurdon Institute, Cambridge, UK) over 10 years ago, whereas BT-549 cells were a gift by Carlos Caldas (CRUK CI, Cambridge, UK). Identities of these cells lines were confirmed by STR genotyping. Our original stock of DT40 cells was obtained from Julian Sale (MRC-LMB, Cambridge, UK) over 10 years ago. Dmel2 cells from David Glover (University of Cambridge, UK) were cultured in Serum free medium (GIBCO) with 110 U ml^−1^ penicillin, 10 mg ml^−1^ streptomycin. CytochalasinD (Sigma-Aldrich) was used at 1 μg ml^−1^. To obtain mitotic extracts of HeLa cells, 9 μM RO3306 was added for 20 h (h), then washed three times and incubated for 15 min.

### Homologous gene targeting in DT40 cells

Gene targeting was performed according to standard protocol^38^. Briefly, homology arms were cloned into pJET or PGEMT-Easy and subcloned into pBluescript II SK^−^ (pSK). The primers used to amplify homology arms of each construct are listed in Supplementary Table 2. A drug resistance cassette (neomycin/Neo, blasticidin/Blasti or puromycin/Puro) was cloned into pSK between BamHI sites^65^. The two alleles of *HSET* and *CEP215* were targeted sequentially: for HSET^KO^, the first allele was targeted with blasticidin and the second with puromycin. For CEP215, the first allele was targeted with neomycin and the second with blasticidin. All final constructs were linearized and transfected as described previously^27^. Targeted integration of the resistance cassettes was screened by PCR. Primers used for PCR reactions are listed in the Supplementary Table 3. To generate CEP215^ΔHBR^ cell lines, CEP215^ΔN^ was subjected to cre recombinase-mediated excision of the antibiotic resistance cassette^27^. This was further targeted to remove exon 12, subjected to another round of cre-mediated excision of antibiotic resistance cassettes. C-terminal TAP tagging of CEP215 was performed as described previously^27^. CEP215-TAP cells were shown to display normal mitotic spindle morphology. For random integration of GFP-HSET and GFP-HSET^N593K^, 10 μg of linearized plasmid was electroporated into HSET^KO^ cells using a gene pulser (Bio-Rad Laboratories) at 250 V and 950 μF. Cells were plated into three 96-well plates and selected by 1.5 mg ml^−1^ neomycin. Drug-resistant colonies were selected and screened for the expression of GFP-tagged proteins. mRNA was isolated using RNAeasy minikit (Qiagen). One microgram of total RNA was reverse transcribed using Super Script II reverse transcriptase and used for PCR analysis.

### Plasmid constructs and transfection in mammalian cells

For testing interactions in Fig. 2, different fragments of human HSET and CEP215 were cloned into pcDNA6-Bioease vectors using Gateway technology (Life Technologies). Primers used to clone into pDONR221 are listed in Supplementary Table 3. Positive clones from pDONR221 were exchanged into Bioease for affinity purifications and MBP and GST for recombinant protein production in bacteria. Primers used to generate Flag-CEP215(ΔHBR) construct are listed in Supplementary Table 3. Flag-CEP215 (ref. 27) was used as template for Q5 site-directed mutagenesis kit (NEB) to introduce deletion of aa500–700 (HBR) of CEP215. Stable cells expressing Flag, Flag-CEP215 and Flag-CEP215(ΔHBR), 1.5 × 10^6^ N1E-115 cells were generated by selecting transfected cells in 96-well plates containing 0.5 mg ml^−1^ Neomycin. After 10–12 days, colonies were picked and screened for the expression of Flag-tagged proteins. HuSH pRS plasmids-encoding CEP215 shRNA or control shRNA (Origene technologies) were transfected in ecotropic Phoenix cells by the calcium phosphate method, and viral supernatants were collected 48 h after transfection and were added to N1E-115 cells (1:1 ratio of carrier to target cells). Polybrene was added to 5 μg ml^−1^ and 72 h after infection of cells, depletion of CEP215 was assessed by immunoblotting. CEP215 (AM16708, Life Technologies) and HSET siRNAs (AM51331, Life Technologies) were transfected using Lipofectamine RNAiMax following manufacturer's instructions. After 72 h of transfection, depletion of the respective proteins was assessed by immunoblotting. Patient B lymphocytes were isolated from blood of affected patient and parent and were immortalized by EBV transformation^36^.

### Yeast two-hybrid assay

Yeast two-hybrid analysis was performed using Gateway-based yeast two-hybrid system. Briefly, truncations of CEP215 and HSET were cloned into PDEST 32 (bait-GAL4 DNA binding domain) or PDEST22 (prey-DNA activation domain) vectors, transformed into yeast and analysed for growth in medium lacking histidine (SCTLH−) supplemented with 50 mM 3-aminotriazole (3AT). Growth in SCTLH− in the presence of 3AT indicates an interaction between proteins that are fused to activation domain and binding domain.

### Recombinant proteins and HSET antibody generation

Recombinant proteins of different truncations of human HSET and CEP215 were cloned using GST and MBP vectors using Gateway technology. Primers used are listed in Supplementary Table 3. Proteins were induced with 1 mM IPTG and purified using Glutathione Sepharose (GE Healthcare) or Amylose resin (NEB) as described earlier^66^. Antibodies were raised in rabbits against bacterially expressed and purified glutathione S-transferase fusion proteins that contained aa300–673 of the human HSET protein. Antibodies were produced by Eurogentec and were subsequently affinity purified against fusion proteins for use in western blotting in chicken. Additional affinity purification against aa625–673 of HSET was carried out for use in immunostainings in chicken.

### Surface plasmon resonance

The binding of the HSET truncations to MBP-tagged CEP215 truncations was determined using the SPR-based biosensor BiacoreT200 (Biacore). Experiments were performed in 10 mM HEPES pH 7.4, 150 mM NaCl, 1 mM EDTA, 0.5% (v/v) Tween-20 at 25 °C. About 1000 RUs of each of the MBP-CEP215 truncations was immobilized on test flow cells (Fc-3, Fc-4) of a CM5 sensor chip using amine-coupling chemistry and non-immobilized flow cell (Fc-1) served as the control flow cell and (Fc-2) was MBP protein alone. One micromolar of each of the truncations was flown over the chip at 30 μl min^−1^ for 120 s and dissociation was followed for an additional 180 s. The chip was regenerated by injecting brief pulses of 0.2 M sodium carbonate, pH 9.5. Data obtained for the control flow cell were subtracted from those obtained for test flow cell and binding evaluated using BIAevaluation software.

### Antibodies and immunostainings

Primary antibodies used in this study were CEP215 (ref. 27, 1:700 or Bethyl laboratories A300–554A 1:500), FLAG (Cell Signaling #2368 1:1000 or Sigma-Aldrich F3165 1:2000); HSET (Bethyl laboratories A300-952A 1:1000 or our own 1:500); centrin-1 (Sigma-Aldrich C7736 1:500); centrin-2 (Biolegend poly6288 1:300); centrin-3 (Abnova H00001070-M01 1:500); Streptavidin HRP (Cell Signaling #3999 1:1000); PCM1 (Abcam ab154142 1:1000); PLK1 (BD biosciences #558446 1:1000); CEP63 (ref. 36), α-tubulin (Dm1α T9026 1:1000 or Dm1α-FITC F2168 1:500 both Sigma-Aldrich); γ-tubulin (GTU88; Sigma-Aldrich 1:1000); dynein intermediate chain (DIC; Abcam ab23905 1:1000) and p150 dynactin (BD Biosciences 610473 1:2000). DNA was stained with Hoechst 33258 (Sigma-Aldrich). DT40 and B cells were processed as described in (ref. 36).

### Affinity purification and immunoprecipitation

For affinity purification of CEP215-TAP complexes, 2 × 10^9^ cells were pelleted and lysed in 5 ml of lysis buffer containing 10 mM Tris-HCl (pH8), 100 mM KCl, 1.5 mM MgCl_2_, 0.5% Triton-X 100, 5% Glycerol and 10 μM β-mercaptoethanol supplemented with protease inhibitor cocktail (Sigma-Aldrich). Cleared whole-cell extracts were obtained by centrifuging cell lysates at 16,000*g* for 15 min at 4 °C and incubated with 200 μl of Streptavidin Dynabeads. After 3 washes with lysis buffer containing 0.2% Triton-X-100 and 3 washes with 25 mM ammonium bicarbonate, samples were subjected to tryptic digestion and mass spectrometry or western blot analysis. Lysates prepared as above were subjected to immunoprecipitation with Dynabead coupled CEP215 antibody for 4 h as described^66^ and processed for western blotting.

### Western blotting and intensity measurements

Whole-cell extracts for western blotting were prepared by lysing cells in RIPA buffer (50 mM Tris, pH 8.0, 150 mM NaCl, 1.0% NP-40, 0.5% sodium deoxycholate, 0.1% SDS and protease inhibitor cocktail). Lysates were separated on 3–8% Tris-acetate or 4–12% Bis-Tris SDS–polyacrylamide gel electrophoresis gels (Life Technologies) and transferred onto nitrocellulose for western blot analysis. Image J was used to quantify signal intensities normalized against appropriate loading controls. Full scans of western blots are included in Supplementary Figs 11–17.

### Sucrose gradient ultracentrifugation and gel filtration

CEP215-TAP-tagged complexes were purified as described before from 5 × 10^9^ cells and eluted in 800 μl of 2 mM Biotin. This was layered onto a 5 to 40% (w/v) continuous sucrose gradient prepared in the lysis buffer minus detergent (900 μl each) and layered onto a 60% w/w cushion. The complexes were then loaded on the gradient and subjected to ultracentrifugation using SW40Ti rotor at 46,600*g* for 16 h at 4 °C. Three hundred microlitre fractions were collected from bottom and TCA-precipitated before being subjected to western blot analyses. Cytoplasmic extracts prepared as above were subjected to gel filtration analysis on a Superose 6 10/300 GL (GE healthcare). High molecular weight kit (Sigma-Aldrich) was used to calibrate the column before analysing samples. Fractions of 250 μl were collected and subjected to Streptavidin affinity purification followed by western blot analysis.

### Centrosome isolation and microtubule nucleation

Centrosomes from the indicated cells were isolated as described^38^. Briefly, cells were treated with 1 μg ml^−1^ cytochalasin D and 3.3 μM nocodazole for 1 h before harvesting. A total of 2 × 10^8^ cells were lysed in hypotonic lysis buffer (1 mM Tris pH 8.0, 0.1% β-mercaptoethanol (freshly added before use), 0.5% NP-40, 0.5 mM MgCl_2_, 150 μl of 20,000 U DNaseI), and centrifuged through a 2 ml 50% w/w sucrose cushion. The cushion-lysate interface (4 ml) was further subjected to a discontinuous gradient sucrose centrifugation (70, 50 and 40% w/w sucrose). Isolated centrosomes from each of the fractions were pelleted through 10 mM PIPES and subjected to western blot analysis. For microtubule nucleation assays, the peak centrosome fractions were pooled and 5 μl of this was added to 20 μl of Xenopus egg extracts and incubated for 10 min and fixed by adding 500 μl of aster fixation solution (BRB80, 10% glycerol, 0.25% glutaraldehyde and 0.1% Triton X-100). This was layered onto a 40% glycerol cushion and centrifuged onto coverslips. The asters were visualized by staining for DM1α-FITC.

### Image acquisition, processing and analysis

Imaging of fixed cells was performed on Nikon Eclipse A1 Ti-E scanning confocal microscope or Leica IR confocal microscope. Images shown here represent 3D projections of z-sections taken every 0.3 μm across the cell. Images represented as a single experiment were acquired using the same settings and were imported into Volocity (6.3; PerkinElmer) and Photoshop (CS6; Adobe) and were adjusted to use the full range of pixel intensities. Super-resolution microscopy was carried out using a Structured Illumination Microscope (SIM) by API OMX Deltavision. Cells were imaged with 100 × 1.4 numerical aperture Olympus objective. Data was reconstructed using API SoftWorx software. For time-lapse imaging of DT40 cells expressing GFP-EB3 cells were settled onto concanavalin A-coated glass bottom dishes (Mat Tek). Cells were kept at 40 °C in a humidified incubation chamber (Tokai) with 5% CO_2_ and were imaged using a spinning-disc confocal system (PerkinElmer) equipped with an electron microscopy charge-coupled device digital camera (C9100-13; Hamamatsu Photonics mounted on an inverted microscope (Eclipse TE2000-S; Nikon). Imaging was carried out with a frame rate of 5 min with z-steps of 1.5 μm using Volocity 2D. N1E-115 and BT-549 cells were seeded into Ibidi 8 well chamber dish and imaging was conducted every 5 min in a humidified chamber with 37 °C and 5% CO_2_, using a Nikon Eclipse TE2000-E microscope, and analysed with NIS-Elements software (Nikon).

HSET levels at centrosomes were determined by measuring mean fluorescence intensity of HSET in γ-tubulin-positive volumes of mitotic cells. Volumes were selected in an automated fashion by applying appropriate intensity thresholding in Volocity 6.3 (PerkinElmer). Identical settings were used on all cells from one experiment regardless of genotype. In the dot plot each dot corresponds to a cell, because in each cell we averaged the mean fluorescence intensity of HSET obtained from the two centrosomes.

For spindle angles, cells were selected in which the two spindle poles fell within 1.8 μm in z (that is, maximum 6 z-steps). Based on intensity thresholding of α-tubulin staining, the centroids of opposite spindle poles were identified by Volocity 6.3 and connected by a line (providing the spindle axis) in an automated fashion. Maximum projections showing the spindle axis were exported into Adobe Illustrator, where position of each centrosome with respect to this axis was determined. For calculating centrosome distance from spindle poles, the centroid of centrosomes (γ-tubulin staining) and the back edge of spindle poles (α-tubulin staining; longest axis points) were identified using intensity thresholding in Volocity 6.3. Coordinates of these points were exported to MATLAB, where distances between centrosome centroid and back edge of pole were calculated.

### Microtubule pelleting assay

DT40 extracts from WT-TAP, CEP215(ΔHBR)-TAP and CEP215(ΔCM1)-TAP were lysed in a buffer containing 50 mM Tris-HCl, pH 7.4, 5 mM MgCl_2_, 0.1 mM EGTA, and 0.5% Triton X-100, supplemented with protease and phosphatase inhibitor cocktails, and passed through a 26-Gauge needle 10 times. Extracts were precleared at 67,700*g* in an MLA130 rotor for 20 min at 4 °C. After addition of 0.5 mM MgGTP and 2 mM MgATP, extracts were warmed to room temperature before sequential addition of 5 and 15 μM taxol. Around 2 mg ml^−1^ of these extracts were mixed with taxol-stabilized microtutubules (0.2 mg ml^−1^) or nontaxol-treated tubulin (0.2 mg ml^−1^) and incubated at 30 °C for 30 min before layering onto a 1 M sucrose cushion in BRB80 buffer (80 mM Pipes, pH 6.8, 1 mM MgCl_2_, and 1 mM EGTA) supplemented with 0.5 mM ATP and with or without 10 μM taxol. Microtubules were pelleted at 67,700*g* in MLA130 rotor for 20 min at 22 °C. Supernatants were saved for immunoblotting. Pellets were washed twice in BRB80 and re-suspended in 1 × SDS–PAGE loading buffer to one fifth of the volume of supernatant. Equal volume of pellets and supernatants were loaded on gel. In the case of MBP-CEP215 (1–300), a total of 500 ng of dialyzed protein in PBS was incubated with microtubules or tubulin and pelleting carried out as before.

### Cell viability assay

To assess cell survival following sh/siRNA, 1 × 10^5^ cells were seeded in a 48-well plate and subjected to shRNA/siRNA treatments. Six days post transfection, CellTiter-Glo substrate was added to cells as recommended by the manufacturer (Promega) and after 10 min of incubation transferred to standard opaque 96-well standard plate and luminescence assayed using PHERAStar.

### NanoLC–MS/MS analysis and data processing

Bead-bound proteins were digested by the addition of 10 μl trypsin solution 15 ng μl^−1^ (Roche) in 100 mM ammonium bicarbonate. The beads were then incubated at 37 °C overnight. A second step digestion was performed the following day for 4 h. Sample tubes were placed on a magnetic rack and the supernatant solution was collected and acidified by the addition of 2 μl 5% formic acid. The samples were then cleaned using Ultra-Micro C18 Spin Columns (Harvard Apparatus) prior to the mass spectrometry (MS) analysis according to manufacturer's instructions. The liquid chromatography–MS (LC–MS) analysis was performed on the Dionex Ultimate 3,000 UHPLC system coupled with the Orbitrap Velos mass spectrometer (Thermo Scientific). Digested peptides were re-suspended in 30 μl of 0.1% Formic acid for injection and a 5 μl volume was loaded on the Acclaim PepMap 100, 100 μm × 2 cm C18, 5 μm, 100 Å trapping column with the μlPickUp Injection mode using the loading pump at 7 μl min^−1^ flow rate for 10 min. For the analytical separation the Acclaim PepMap RSLC, 75 μm × 25 cm, nanoViper, C18, 2 μm, 100 Å column retrofitted to the nanospray source was used for multi-step gradient elution. Solvent A was composed of 0.1% formic Acid, 2% MeCN and 5% DMSO with and solvent B was composed of 80% acetonitrile, 0.1% formic acid, 5% DMSO. The gradient elution method at flow rate 300 nl min^−1^ was as follows: for 60 min gradient up to 45% (B), for 10 min gradient up to 95% (B), for 10 min isocratic 95% (B), for 5 min down to 5% (B), for 10 min isocratic equilibration 5% (B) at 40 °C. Separated peptides were transferred to the gaseous phase with positive ion electrospray ionization applying a voltage of 2.0 kV. Targeted ions already selected for MS/MS were dynamically excluded for 40 s. Top 20 multiply charged precursor isotopic clusters with m z^−1^ value between 400 and 1,600 m z^−1^ were selected with FT mass resolution 60K and isolated for CID fragmentation within a mass window of 2.0 m z^−1^ and collision energy 28. The CID tandem mass spectra were processed using the SequestHT and Mascot search engines implemented on the Proteome Discoverer software version 1.4 for peptide and protein identifications. All spectra were searched against a UniProtKB/Swiss-Prot and UniProtKB/TrEMBL fasta file. The Nodes for SequestHT and Mascot included the following parameters: Precursor Mass Tolerance 10 p.p.m., Fragment Mass Tolerance 0.5 Da, Dynamic Modifications were Oxidation of M (+15.995 Da) and Deamidation of N, Q (+0.984 Da). The level of confidence for peptide identifications was estimated using the Percolator node with decoy database search. FDR<1% was applied in all the experiments.

For network construction, we performed the following workflow. We extracted a non-redundant list of interactors identified from pulldowns of CEP215-TAP cells that were present in at least 2 experiments. To minimize non-specific binders (that is, proteins that bind streptavidin beads or the TAP tag) we removed proteins that were represented even by a single peptide in pulldowns from untagged WT cells and other TAP-tagged cell lines generated in the group such as TAP-CEP63 and TAP-CEP135. Next, we used this dataset to screen for proteins represented by at least 4 unique peptides in 2 experiments (worksheet Filtered_2 in Supplementary Table 2). Finally, based on the Filtered_2 dataset, we shortlisted proteins present in all three experiments and included these in the worksheet called Filtered_3 in Supplementary Table 2. To represent our final network, we shortlisted 23 proteins using GO analysis, excluding proteins limited to nucleus, spliceosome or membrane in their localization (Supplementary Table 1).

### Sequence orthology detection and conservation analysis

Sequence orthologues of CEP215 and HSET were obtained from OMA orthology database and EnsemblCompara, which adopt complementary approaches for sequence orthology detection. We considered only one-to-one orthologues and disregarded any paralogues (gene-duplicates). OMA detects orthologues using an inference algorithm, which first infers homologous sequences by performing all-against-all Smith-Waterman alignments between all sequences and retain significant matches. Subsequently, orthologous pairs (the subset of homologues related by speciation events) were inferred using mutually closest homologues based on evolutionary distances, taking into account distance inference uncertainty and the possibility of hidden paralogy due to differential gene losses. On the other hand, EnsemblCompara uses maximum likelihood phylogenetic gene trees obtained from the protein-based multiple alignments and reconciles them with established species tree and permits duplication calls on internal nodes. In addition, we have also included experimentally determined homologues of CEP215 (centrosomin/cnn in fruitfly and Mto1 and pcp1 in fission yeast)^67^,^68^ and HSET (Kar3 family protein pkl in fission yeast)^16^. Sequence alignments for CEP215-HBR and HSET aa1–150 were generated using MAFFT from EMBL-EBI web server. Pairwise sequence identity (in percentage) between human HSET aa1–150 and orthologues was estimated using ClustalW server^69^.

### Statistical analyses

Statistical analysis and graphs were carried out using Microscoft Excel or R. The numbers of experimental repeats or cells scored are reported in figures and figure legends. Data are presented as mean±s.d. unless stated otherwise. Statistical test used for each experiment is stated in the legend.

## Additional information

**Accession codes:** Proteomics data have been deposited to the ProteomeXchange Consortium via the PRIDE partner repository with the dataset identifier PXD003382^70^.

**How to cite this article:** Chavali, P. L. *et al*. A CEP215–HSET complex links centrosomes with spindle poles and drives centrosome clustering in cancer. *Nat. Commun.* 7:11005 doi: 10.1038/ncomms11005 (2016).

## Supplementary Material

Supplementary Figures and Supplementary Tables Supplementary Figures 1-17 and Supplementary Tables 1-2

Supplementary Data 1 Unfiltered and filtered proteomics datasets used to derive CEP215 interactome

Supplementary Movie 1 Mitosis in a WT DT40 cell expressing GFP-EB3. Images were acquired at a rate of 5 minutes/frame.

Supplementary Movie 2 Mitosis in a CEP215^ΔHBR^ DT40 cell expressing GFP-EB3. Note partial detachment of centrosomes. Images were acquired at a rate of 5 minutes/frame.

Supplementary Movie 3 Mitosis in a CEP215^ΔHBR^ DT40 cell expressing GFP-EB3. Note complete detachment of centrosomes. Images were acquired at a rate of 5 minutes/frame.

Supplementary Movie 4 Mitosis in a CEP215^ΔHBR^ DT40 cell expressing GFP-EB3. Note complete detachment of centrosomes and abnormal centrosome segregation. Images were acquired at a rate of 5 minutes/frame.

Supplementary Movie 5 Mitosis in a HSET^KO^ DT40 cell expressing GFP-EB3. Note loss of spindle focus and complete detachment of centrosomes. Images were acquired at a rate of 5 minutes/frame.

Supplementary Movie 6 Mitosis in a HSET^KO^ DT40 cell expressing GFP-EB3. Note collapse of spindle into transient monopolar configuration. Images were acquired at a rate of 5 minutes/frame.

Supplementary Movie 7 Multipolar mitosis in a HSET^KO^ DT40 cell expressing GFP-EB3. Images were acquired at a rate of 5 minutes/frame.

Supplementary Movie 8 N1E115 cells transduced with control shRNA. Images were acquired at a rate of 5 minutes/frame.

Supplementary Movie 9 N1E115 cells transduced with shCEP215. Images were acquired at a rate of 5 minutes /frame.
