# Supplementary material for: A CEP215–HSET complex links centrosomes with spindle poles and drives centrosome clustering in cancer
Source: Nat Commun. 2016 Mar 18;7:11005. doi: 10.1038/ncomms11005 (PMC4802056; doi:10.1038/ncomms11005)
Supplement: Supplementary Figures and Supplementary Tables — Supplementary Figures 1-17 and Supplementary Tables 1-2 [file ncomms11005-s1.pdf]

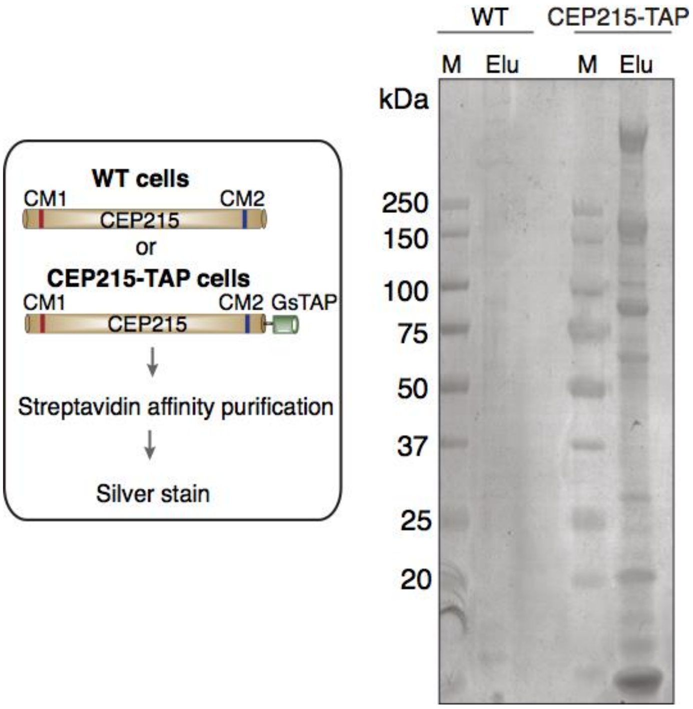

Supplementary Figure 1. Affinity purification of CEP215-bound protein complexes. A silver-stained gel representative of the affinity purification experiment employed to identify the CEP215 interactome in Fig 1. Following biotin elution, 50% of the sample was loaded on a gel and processed for silver staining.

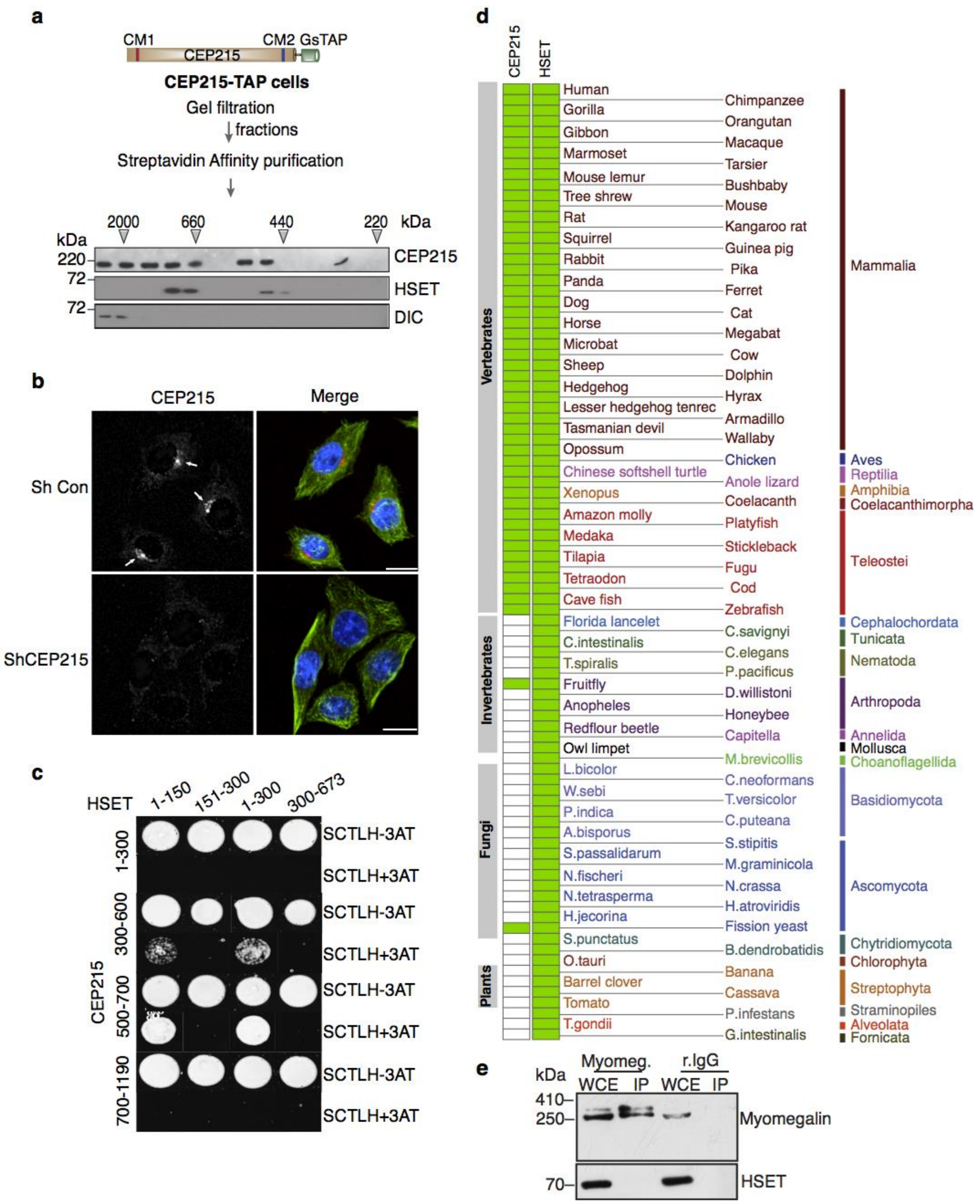

Supplementary Figure 2. The HSET-CEP215 interaction is specific to the vertebrate lineage. a. Workflow used to identify HSET and dynein complex with CEP215 is shown. Cytoplasmic cell extracts of CEP215-TAP cells were subjected to gel filtration followed by affinity purification of complexes from each fraction. Antibodies used for western blotting are indicated. Arrowheads indicate molecular weights, identified by calibration with molecular weight standards. **b.** Images show HeLa cells stably expressing retroviral scrambled or CEP215-targeting ShRNAs (ShCon and ShCEP215, respectively). Cells are stained for

CEP215 (red in merge) and α–tubulin (green in merge). DNA is in blue. **c.** Yeast two-hybrid assay between N-terminal truncations of CEP215 (bait) and truncations of HSET (prey). Positive interactions were screened for growth in the presence of 50 mM 3-amino triazole (3AT). **d.** Orthologs from different organisms screened for conservation of aa1-150 of HSET and HBR of CEP215 (extension to *Fig 2f*). Green cells highlight the presence of orthologs while white cells depict the absence of corresponding sequences (see Methods). **e**. WCE of mitotic HeLa cells were subjected to immunoprecipitation by an anti-Myomegalin antibody or random IgG (con) followed by western blotting with the indicated antibodies.

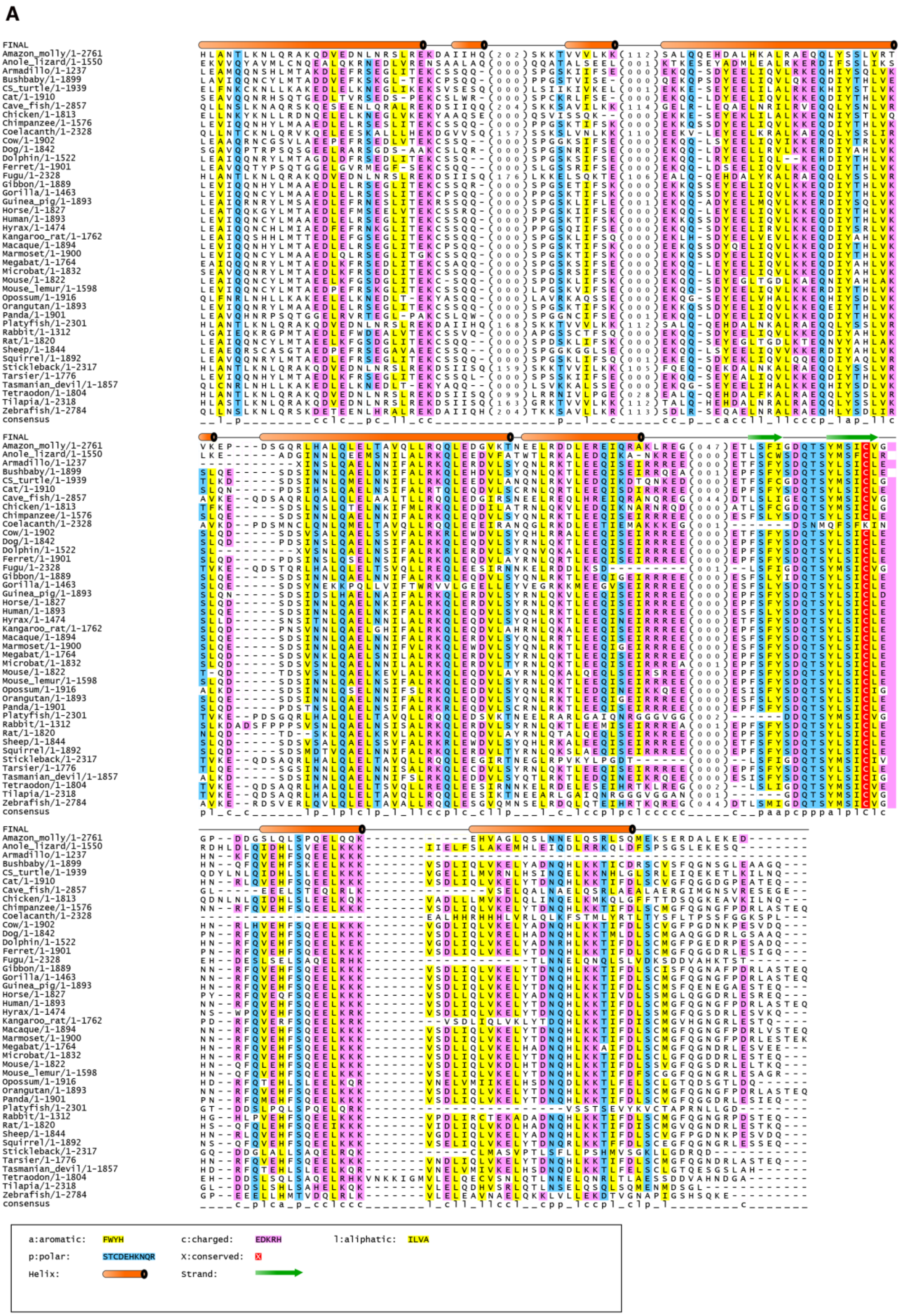

Supplementary Figure 3. Alignment of the HSET binding region (HBR) of CEP215.

Sequence alignment of HBR, corresponding to aa500-700 in human CEP215.

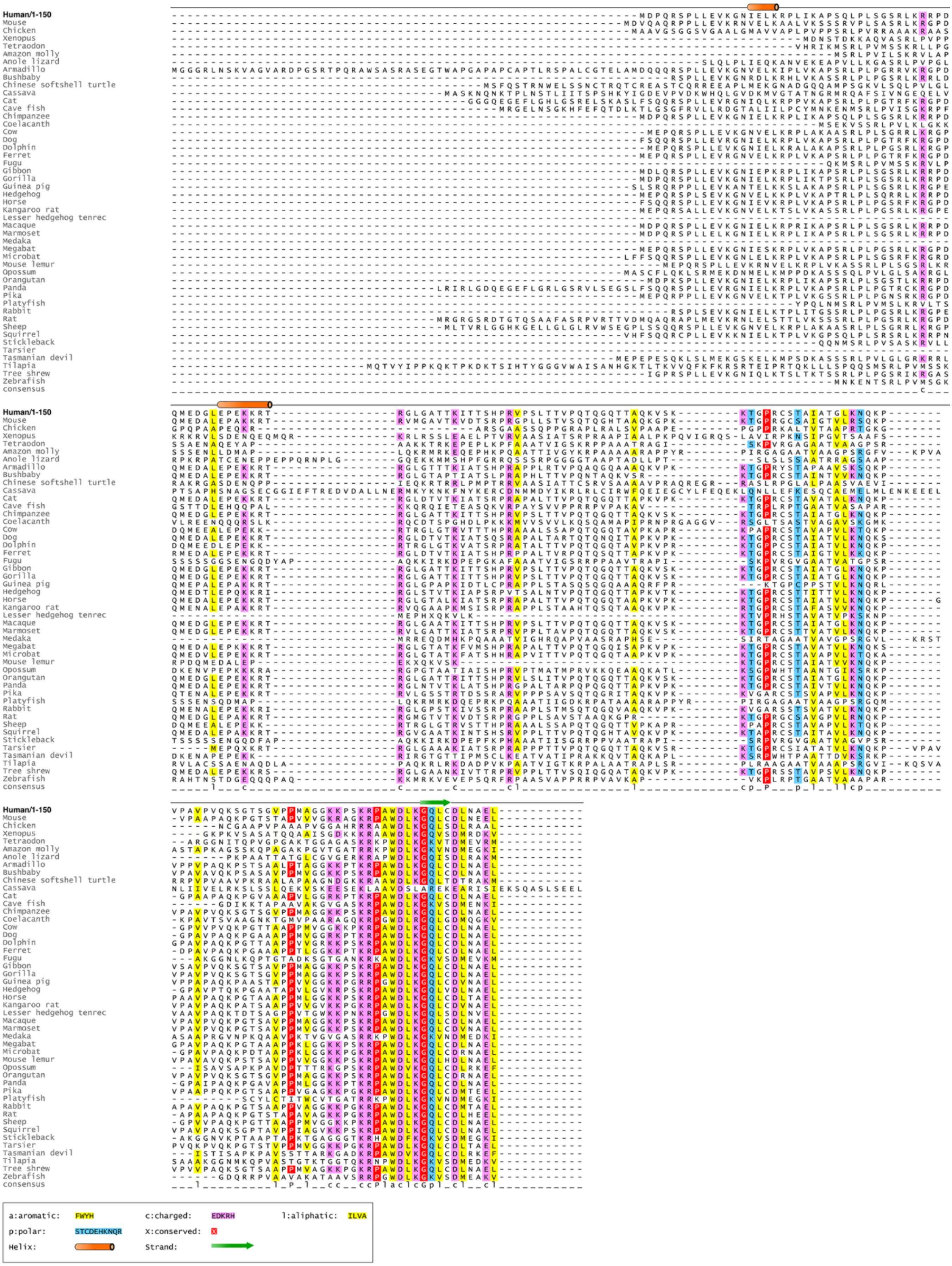

Supplementary Figure 4. Sequence alignment of the CEP215-interacting region of HSET.

This region corresponds to aa1-150.

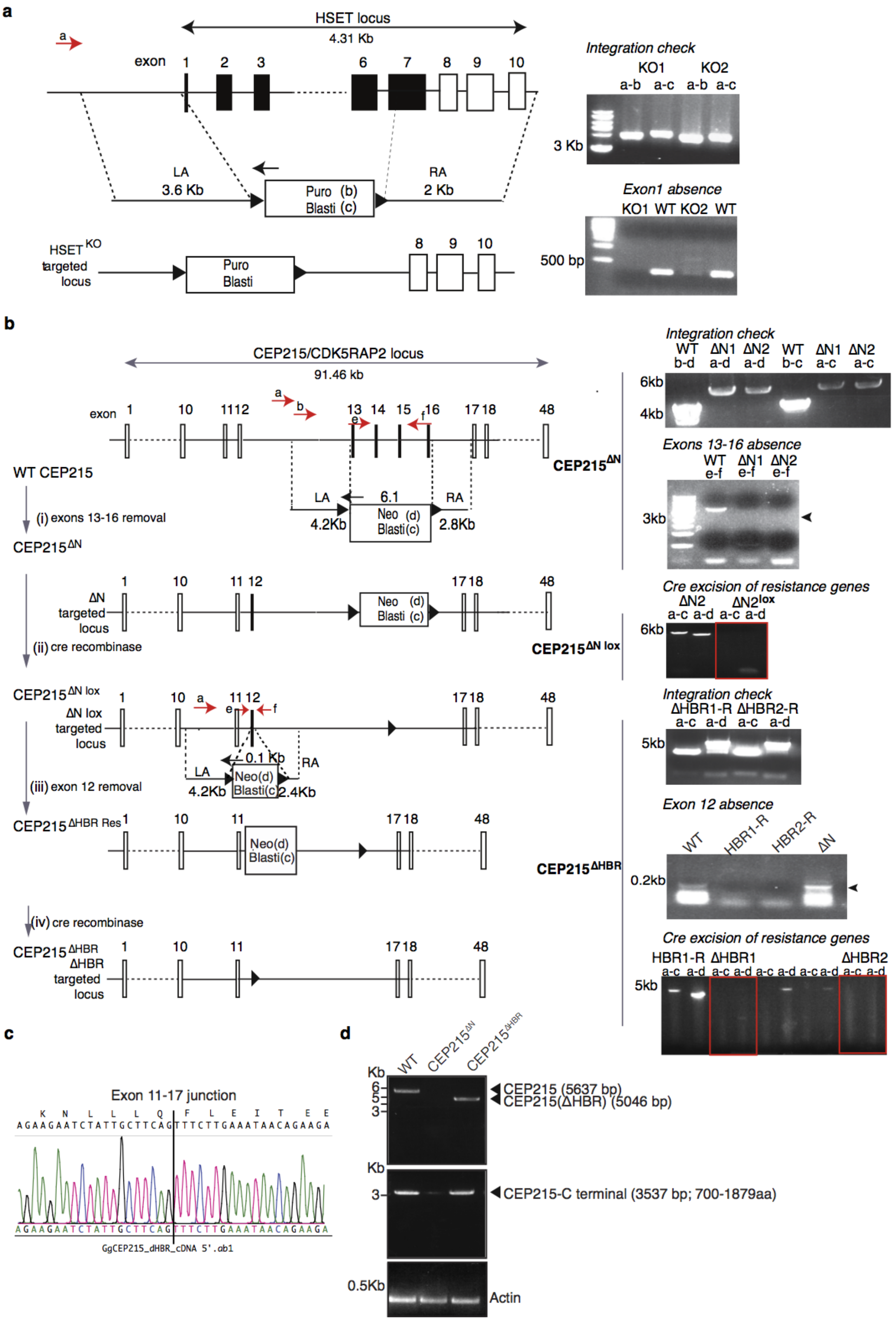

Supplementary Figure 5. Generation of HSET^{KO}, CEP215$^{\Delta HBR}$, CEP215$^{\Delta N}$ DT40 cells. a. Design of homologous gene targeting of *HSET* alleles in DT40 cells. To generate HSET^{KO} alleles, exons 1-7 were replaced by blasticidin and then puromycin resistance genes. Homology arms flanking the targeted region are marked as LA and RA. Using primer position (a) and sequences from the antibiotic resistance cassettes, diagnostic PCR reactions

were used to confirm integration of antibiotic resistance cassettes at correct genomic loci. PCRs of genomic DNA extracted from cells of indicated genotypes are shown. An additional primer that maps within targeted region was used to confirm $HSET^{KO}$ clones. Exon 1 was absent in two different $HSET^{KO}$ clones but present in WT. **b.** Design of homologous gene targeting of CEP215 alleles in DT40 cells. Chicken $CEP215^{\Delta HBR}$ corresponds to aa468-665. Homology arms flanking the targeted region are marked as LA and RA. Locations of primers are marked on the CEP215 locus. $CEP215^{\Delta HBR}$ cells were generated in a 4-step strategy (i-iv). For each step diagnostic PCRs using genomic DNA from relevant cells are shown on right to confirm correct genotypes. First, exons 13-16 of the two CEP215 alleles were sequentially replaced by neomycin- and blasticidin-resistance genes, thereby generating the intermediate $CEP215^{\Delta N}$ cell line (i). Primer pairs a-d and a-c were used to check for the targeted integration of the homologous gene-targeting construct containing neomycin and blasticidin, respectively. Primer pair e-f maps to targeted region (exons 13-16) and the absence of PCR product confirms successful targeting of this region in both alleles of $CEP215^{\Delta N}$ cells. Second, antibiotic resistance genes were removed by cre expression to produce $CEP215^{\Delta N\text{-}lox}$ alleles (ii). Third, removal of exon 12 in $CEP215^{\Delta N\text{-}lox}$ cells generated $CEP215^{\Delta HBR\text{-}R}$ cells (iii). Fourth, these cells were then subjected to cre recombinase-mediated excision of resistance genes to generate the $CEP215^{\Delta HBR}$ alleles (iv). **c.** Confirmation of in-frame HBR deletion (i.e. fusion of exon 11 to 17) by sequencing reverse transcribed CEP215 mRNAs from wild-type and $CEP215^{\Delta HBR}$ cells. **d.** PCRs with primers against exons 1 and 48 and exons 17 and 48 (CEP215 C terminal) of CEP215 were performed mRNAs isolated and reverse transcribed from wild-type, $CEP215^{\Delta N}$ and $CEP215^{\Delta HBR}$ cells.

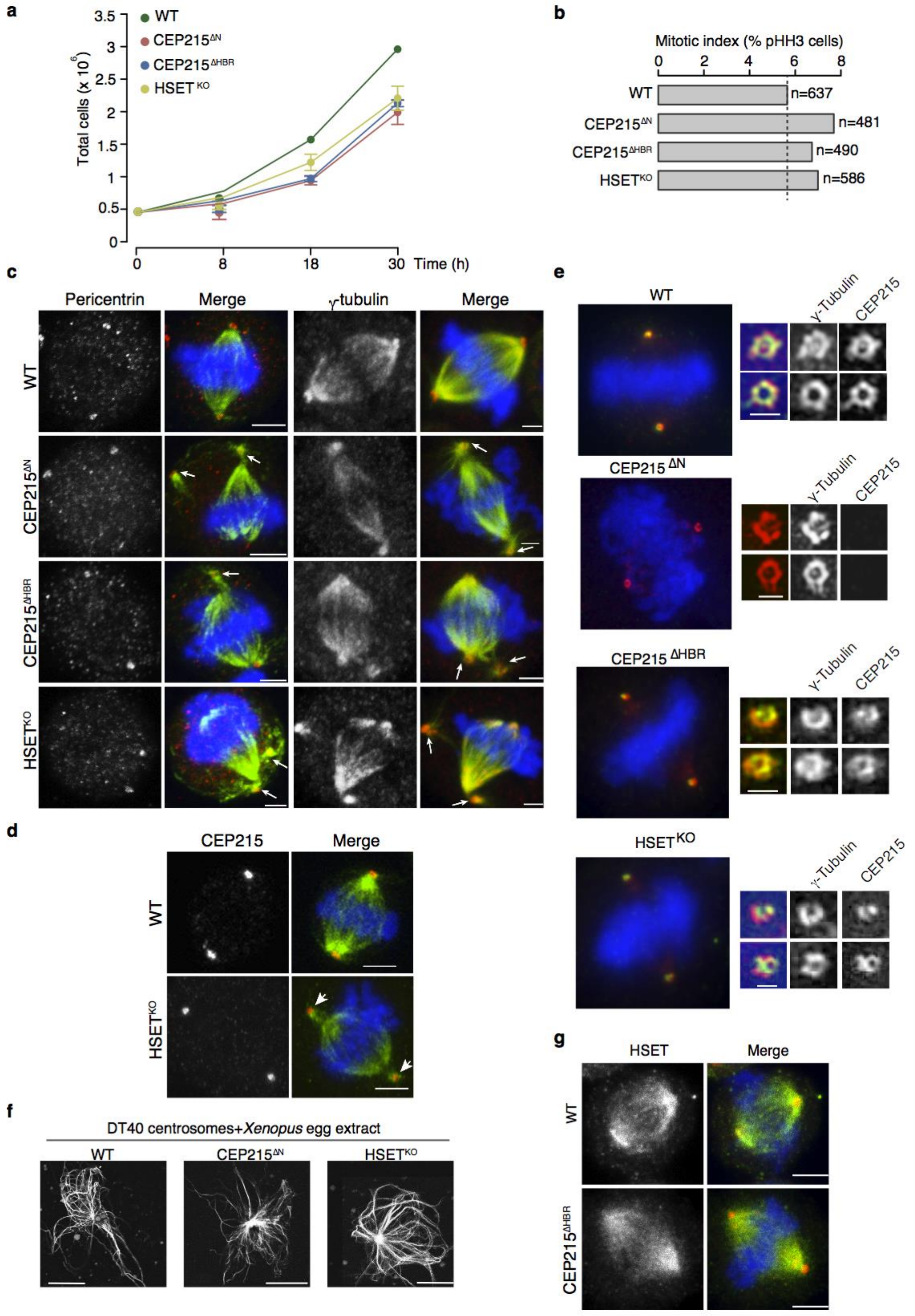

Supplementary Figure 6. Centrosome integrity and nucleation potential are preserved in CEP215$^{\Delta HBR}$, CEP215$^{\Delta N}$ and HSET^{KO} cells. a. CEP215$^{\Delta HBR}$, CEP215$^{\Delta N}$ and HSET^{KO} cells display mild growth impairment. Total number of viable cells over the indicated time period analysed by Vi-Cell. **b.** Graph shows mitotic index of CEP215$^{\Delta HBR}$, CEP215$^{\Delta N}$ and HSET^{KO} scored as percentage of cells with positive phospho-histone H3 staining (pHH3). Total number of cells analysed is indicated. **c.** Images show cells stained for pericentrin (red in merge) or γ-tubulin (red in merge) with α-tubulin (green in merge). DNA is stained blue.

Arrows mark detached centrosomes. Genotypes are indicated on left. **d.** Images show cells stained for CEP215 (red in merge) and α-tubulin (green in merge). DNA is stained blue. Scale bars=3 μm. Genotypes are indicated on left. **e.** 3D structured illumination microscopy (SIM) images of mitotic cells stained for γ-tubulin (red in merge) and CEP215 (green in merge). DNA is stained blue. Ring structures are present in all genotypes. Scale bars=0.5μm. Images represent maximum projections. **f.** Microtubule nucleation potential of centrosomes isolated from WT, CEP215$^{\Delta N}$ and HSET^{KO} cells were assayed using mitotic *Xenopus* egg extracts. Images show examples for centrosomal asters for each genotype. **g**. Images show cells stained for HSET (green in merge) and γ-tubulin (red in merge). DNA is stained blue. Genotypes are indicated on left. Scale bars=3 μm

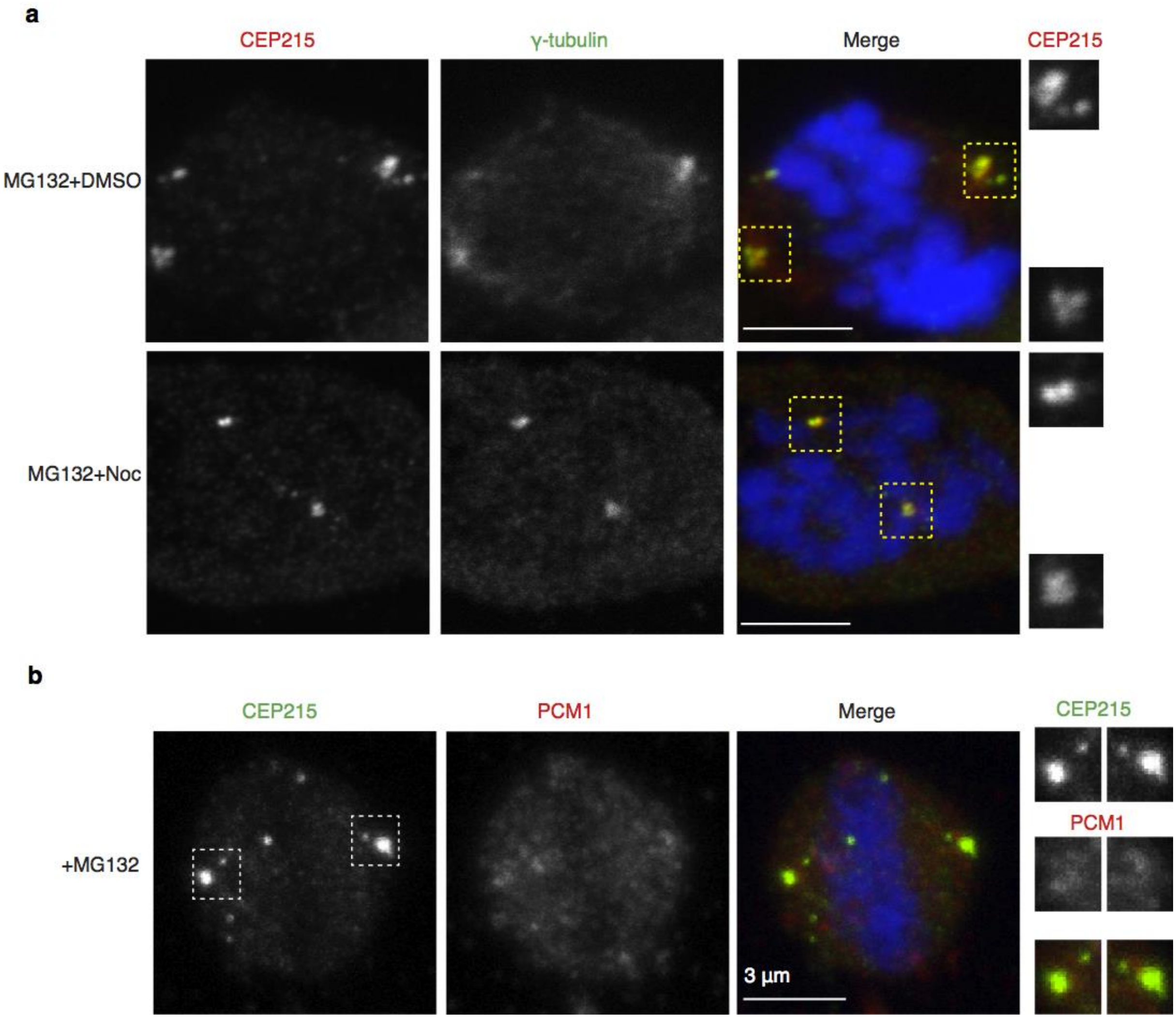

Supplementary Figure 7. CEP215 flares are sensitive to microtubule depolymerisation. a. Representative images show MG132-treated mitotic cells stained for CEP215 (green in merge) and γ-tubulin (red in merge) in DT40 cells following 1-hour treatment with DMSO or nocodazole (1µg/ml). DNA is in blue. Insets show flares of CEP215. Scale bar= 4µm **b.** Images show MG132-treated cells stained for CEP215 (green in merge) and PCM1 (red in merge) in DT40 cells.

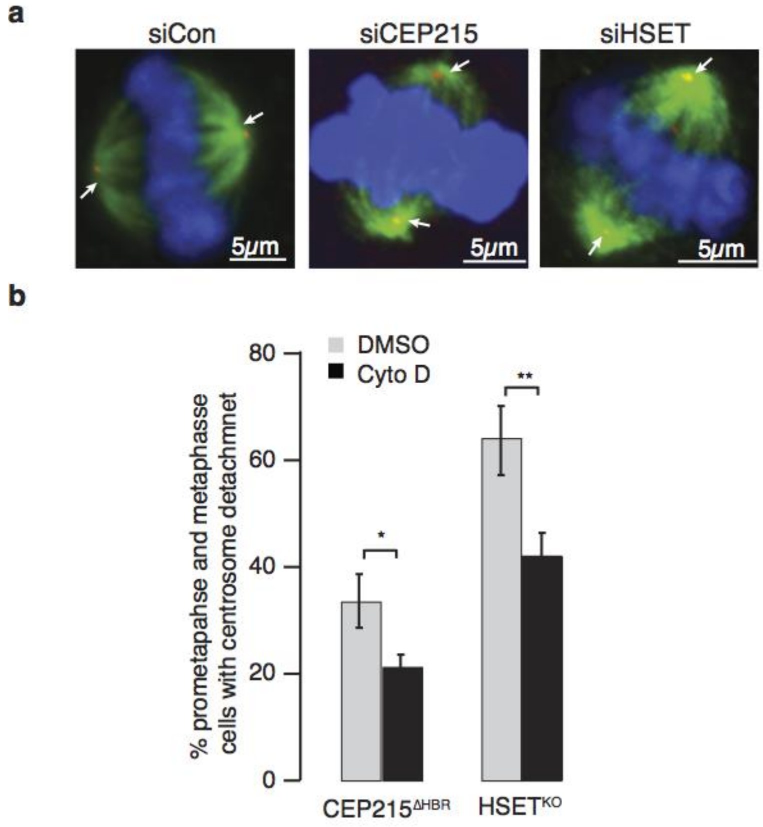

Supplementary Figure 8. Actin depolymerisation partially suppresses centrosome detachment. a. Images depict centrosome positioning in mitotic HeLa cells treated with control (siCon), CEP215 (siCEP215) or HSET (siHSET) siRNAs for 72 hours. Cells were cells stained for the centrosomal marker centrin (red in merge) and the spindle protein, TPX2 (green in merge). DNA is in blue. Arrows indicate centrosomes off-centered with respect to spindle axis. **b.** Graph depicts quantification of centrosome detachment phenotype following 1-hour treatment of WT and CEP215$^{\Delta HBR}$ DT40 cells with DMSO or 1 µg/ml cytochalasin D. Paired t-test (*= p<0.05, **=p<0.005); n=3 biological replicates.

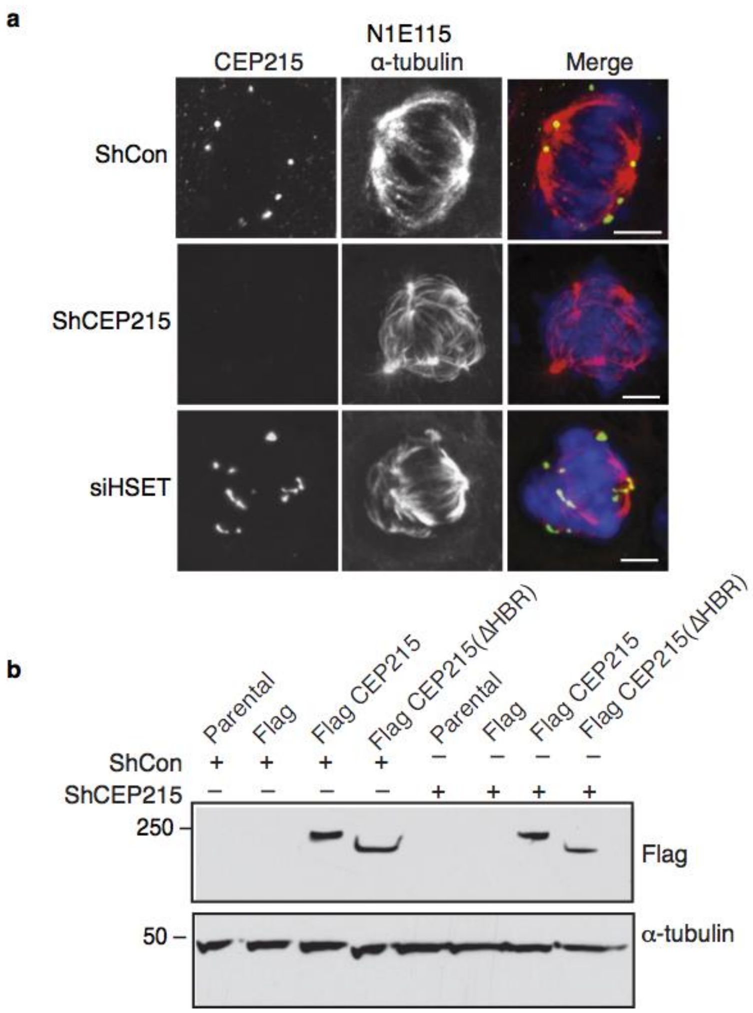

Supplementary Figure 9. CEP215 depletion in N1E115 cells. a. Images show cells treated with control (ShCon) or CEP215 (ShCEP215) shRNAs, or HSET siRNA. Cells were stained for CEP215 (green in merge) and α-tubulin (red in merge). DNA is in blue. Scale bars=8μm **b.** Parental N1E-115, or cells carrying stably integrated Flag, Flag-CEP215 and Flag-CEP215(HBR) constructs, were transduced with control (shCon) or CEP215 (shCEP215) shRNAs. Whole cell extracts were immunblotted for Flag expression.

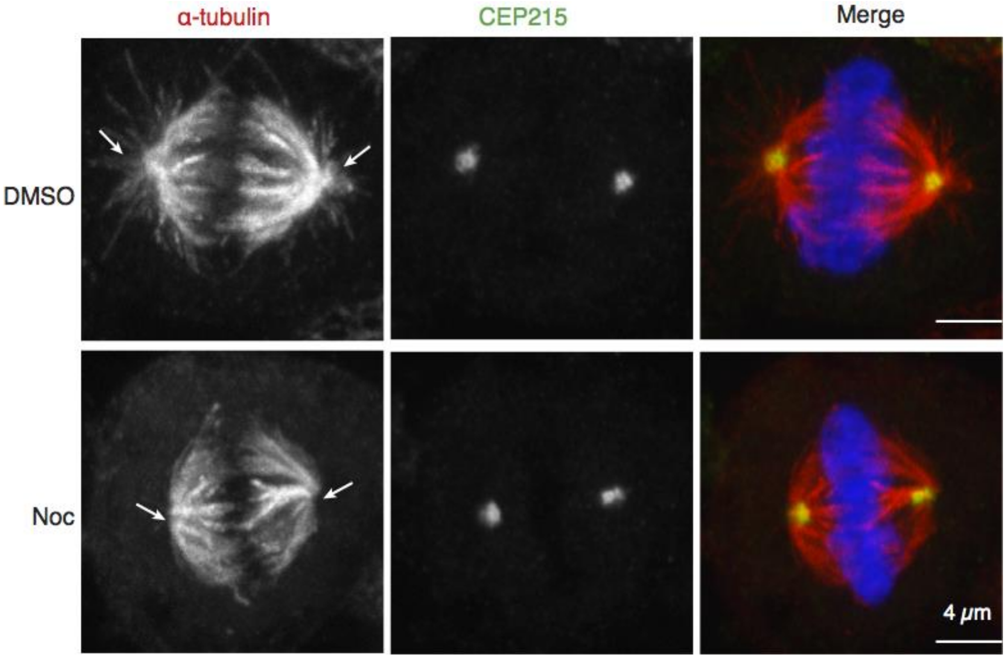

Supplementary Figure 10. Centrosomes are found within spindle poles when astral microtubules are depolymerized. Images show HeLa cells following 10 minute treatment with DMSO or 10nM nocodazole. Cells were stained for CEP215 (green in merge) and α-tubulin (red in merge). DNA is in blue. Arrows mark astral microtubules.

Figure 1c

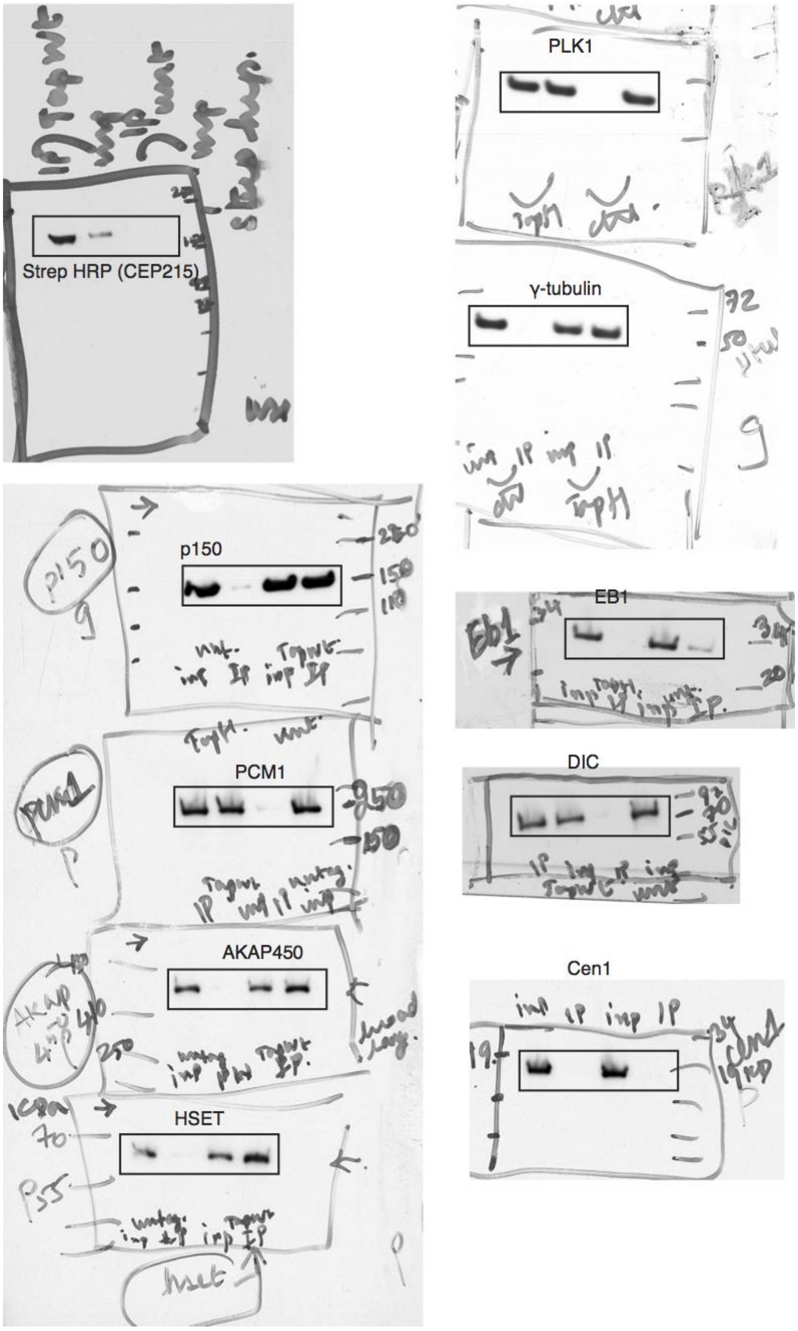

Supplementary Figure 11. Full size scans of blots related to Fig. 1c. The cropped regions are indicated by black rectangles and the antibody used is indicated**.**

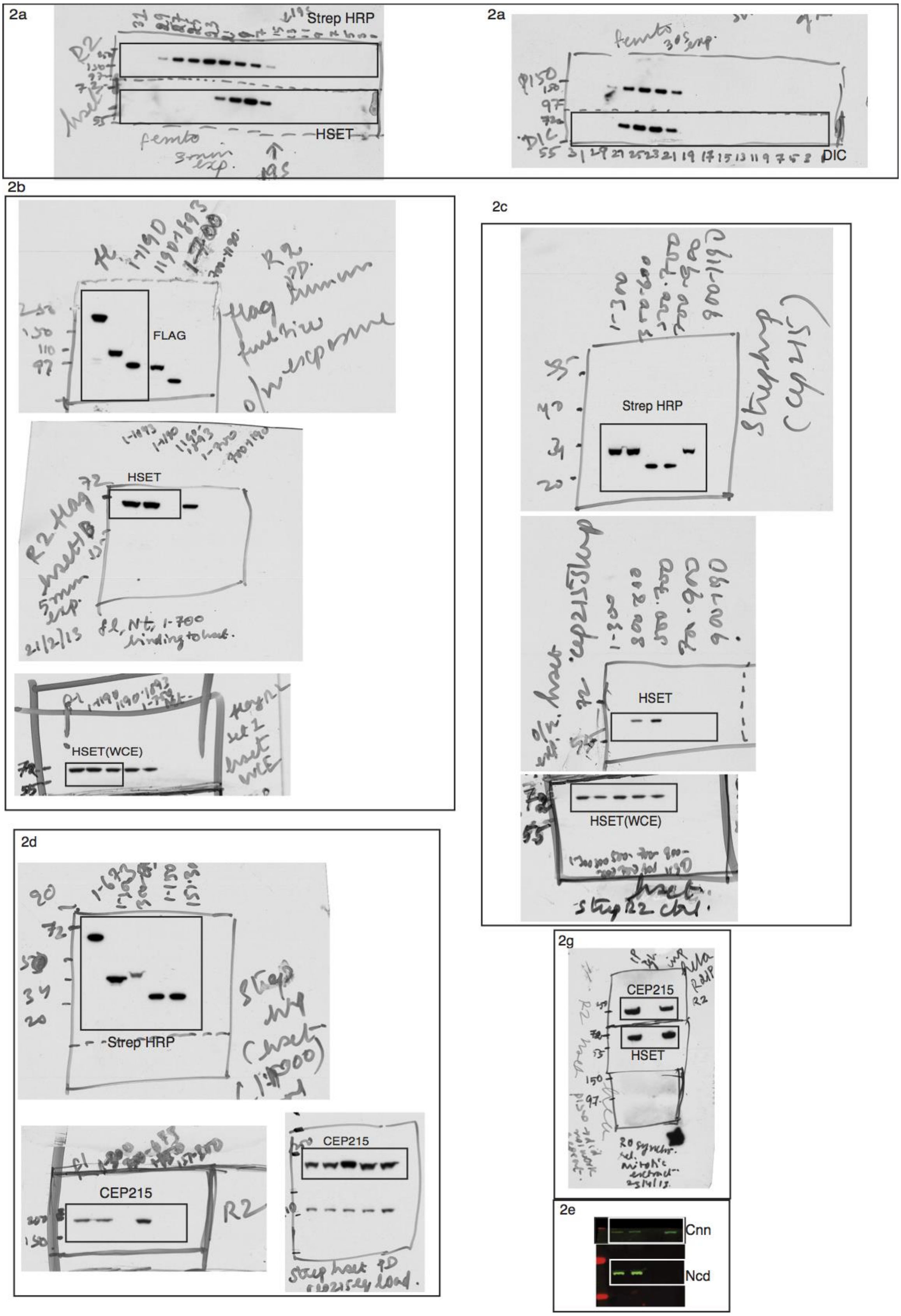

Supplementary Figure 12. Full size scans of blots related to Fig. 2. The cropped regions are indicated by black rectangles and the antibody used is indicated**.**

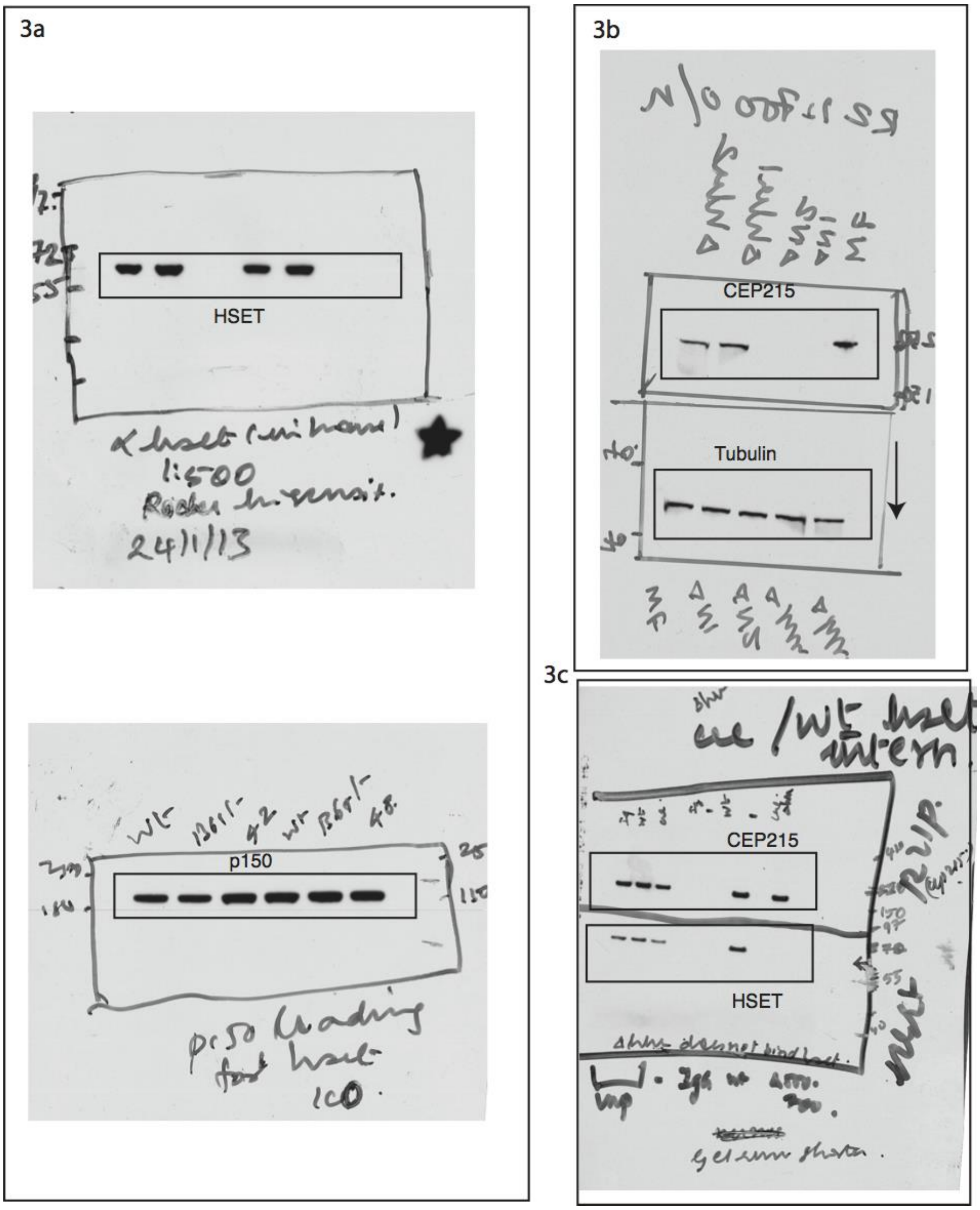

Supplementary Figure 13. Full size scans of blots related to Fig. 3. The cropped regions are indicated by black rectangles and the antibody used is indicated**.** Note that the blot in Fig. 3i is full size.

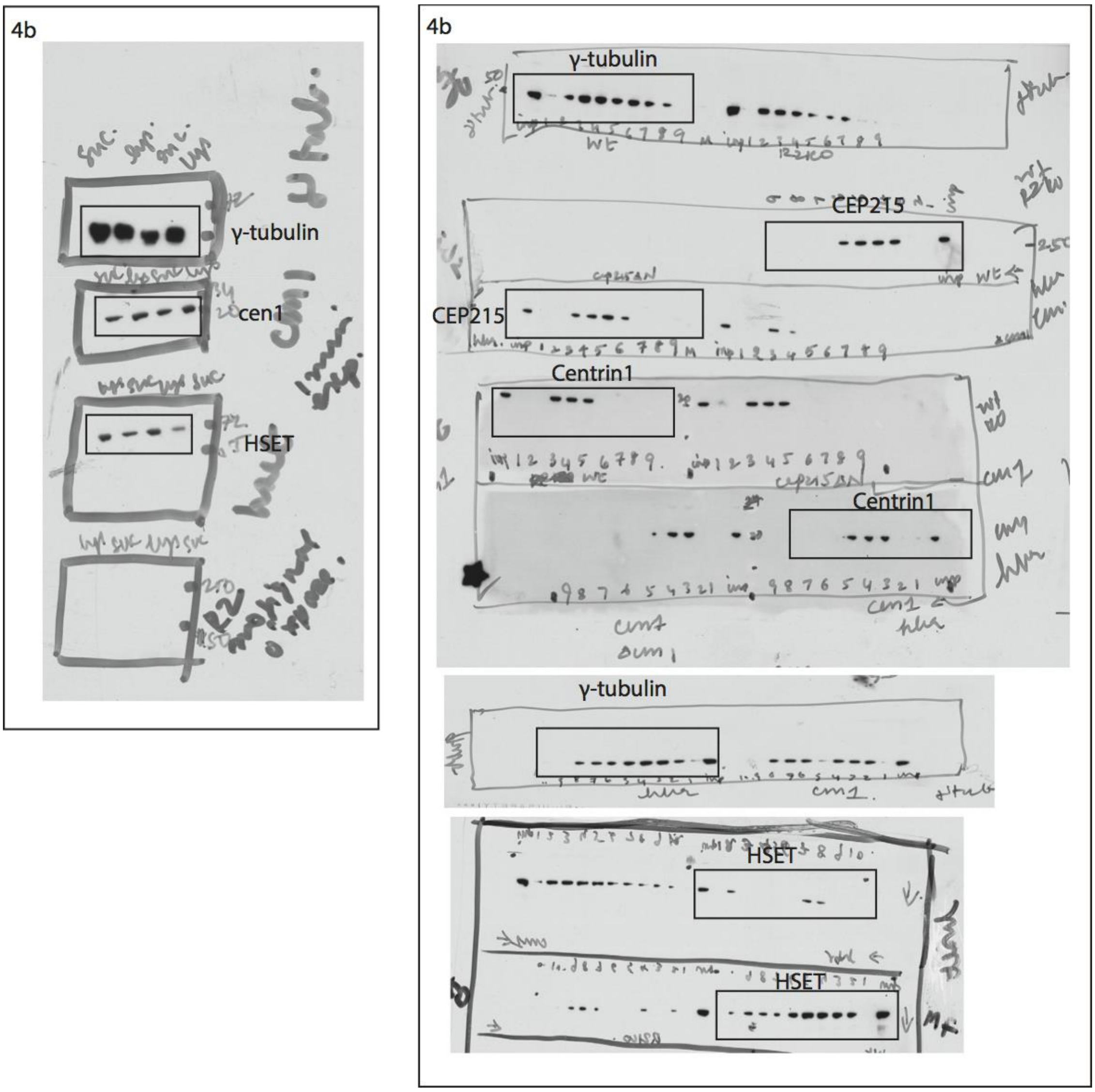

Supplementary Figure 14. Full size scans of blots related to Fig. 4. The cropped regions are indicated by black rectangles and the antibody used is indicated**.**

5b

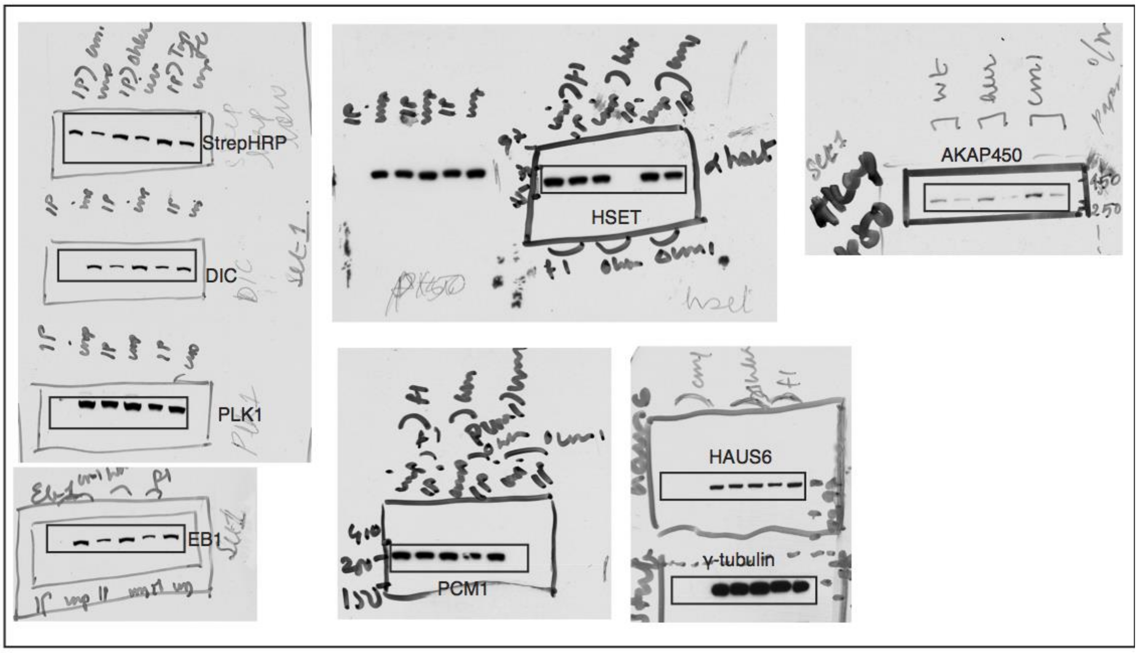

5d

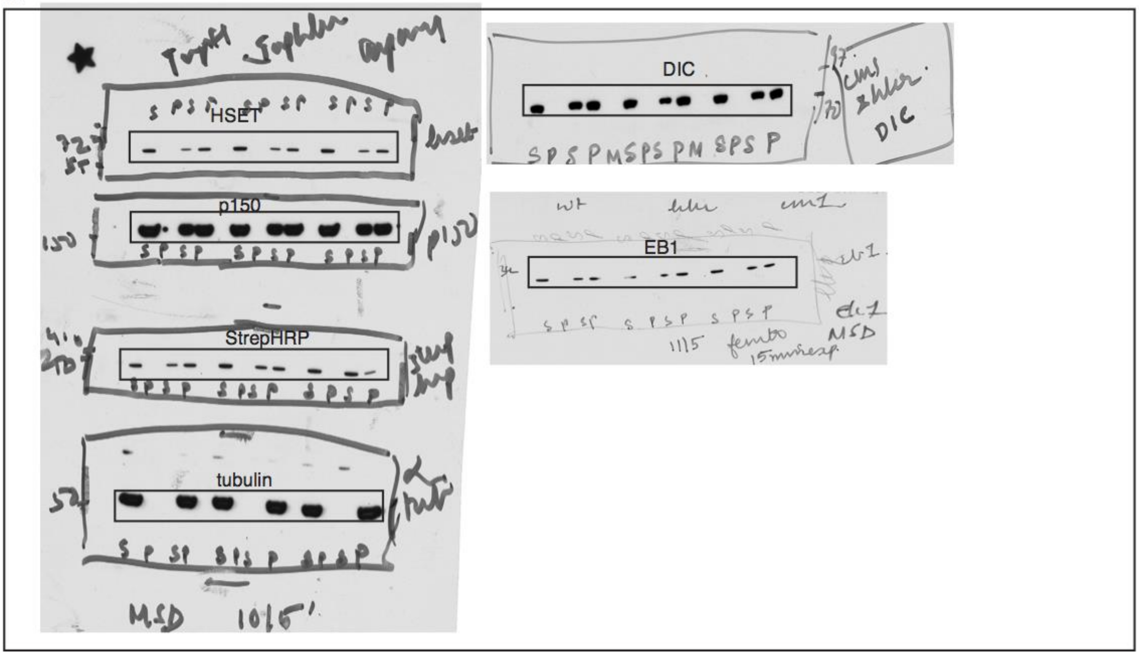

Supplementary Figure 15. Full size scans of blots related to Fig. 5. The cropped regions are indicated by black rectangles and the antibody used is indicated**.** Note that the blot in Fig. 5a is full size.

6b

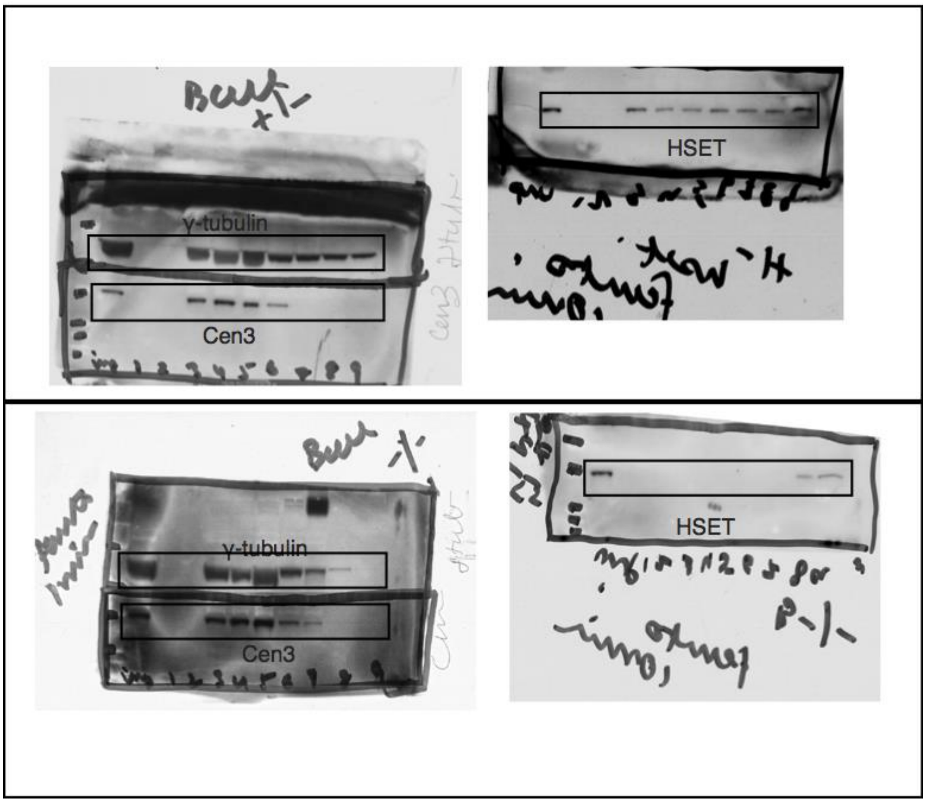

Supplementary Figure 16. Full size scans of blots related to Fig. 6b. The cropped regions are indicated by black rectangles and the antibody used is indicated**.** Note that the blot in Fig. 6a is full size.

7a

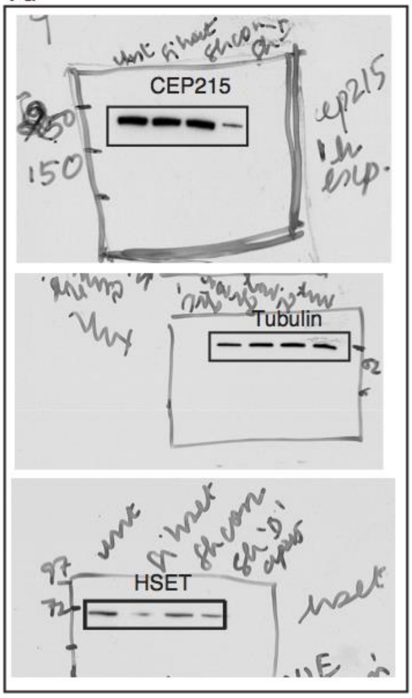

7e

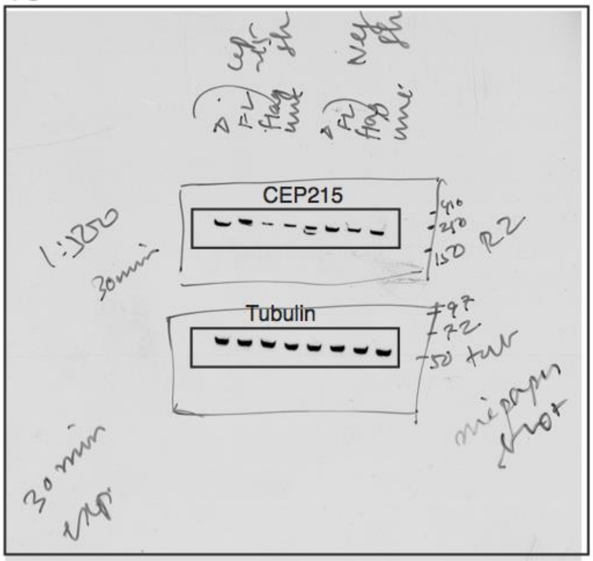

8a

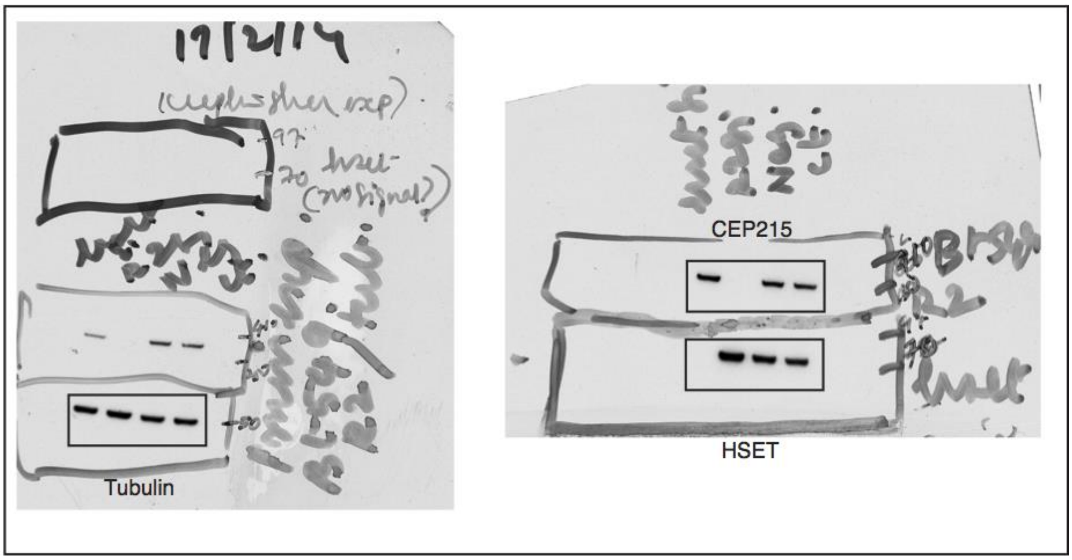

Supplementary Figure 17. Full size scans of blots related to Figs. 7 and 8. The cropped regions are indicated by black rectangles and the antibody used indicated**.**

Accession no.	Protein	Unique peptides (Coverage)			Σ# PSMs		
		Exp1	Exp2	Exp2	PSM1	PSM2	PSM3
**F1NH99**	**CEP215**	**30 (21.61)**	**9 (8.04)**	**69 (41.04)**	**57**	**16**	**184**
A0A023PTR9	HSET	13 (22.51)	6 (15.76)	3 (6.43)	28	14	5
E1BQS8	CCDC77	4 (15.21)	5 (16.67)	2 (6.67)	8	9	4
E1BR81	WDR67	5 (7.77)	4 (5.55)	1 (1.2)	10	7	2
E1BSR3	CLASP2	13 (16.84)	9 (10.92)	1 (1.26)	30	20	2
E1BTL1	AZI1	17 (22.75)	13 (19.27)	9 (13.49)	35	28	18
E1BVH4	SEPT7	5 (18.31)	5 (18.07)	12 (36.39)	13	14	46
E1C062	IST1	5 (22.5)	6 (22.68)	4 (14.11)	16	13	10
E1C245	LOC101750034	15 (4.77)	20 (7.41)	20 (5.3)	31	40	40
E1C3D2	SEPT2	4 (14.09)	6 (24.31)	12 (50.28)	10	11	26
E1C834	PCM1	17 (13.49)	11 (7.6)	21 (13.89)	33	21	45
E1C9H8	PRKAR2A	8 (31.25)	3 (13.5)	14 (51.75)	17	6	44
F1NCX7	PLK1	5 (8.55)	4 (8.1)	3 (8.7)	10	8	6
F1NKL4	DYNC1H1	85 (26.5)	31 (10.73)	15 (4.4)	179	56	30
F1NLX2	SEPT6	6 (26.06)	9 (23.94)	7 (32.39)	15	20	28
F1N9Y0	TUBG1	8 (31.38)	4 (18.03)	4 (14.75)	18	9	8
Q5F3T2	SEPT9	4 (8.05)	8 (21.58)	10 (21.4)	8	19	27
Q5ZK62	ACAP2	10 (23.82)	16 (33.2)	19 (33.93)	31	43	45
Q5ZLC7	EB1	4 (24.81)	1 (6.98)	9 (43.8)	7	2	17
Q9PTG6	DCTN2	8 (26.62)	2 (8.46)	5 (17.66)	16	4	9
Q9W6V0	AKAP9/Hyperion	33 (12.72)	9 (3.51)	148 (47.43)	66	15	362
R4GIN8	PRKACB	4 (18.75)	1 (7.14)	12 (42.56)	8	2	27
R4GJ34	CKAP5	15 (12.09)	22 (16.26)	4 (3.02)	29	48	6

Supplementary Table 1. List of selected candidates used to construct network in Fig. 1b. For each protein the number of unique peptides and the coverage (parentheses) are shown together with the sum of spectral matches obtained from SEQUEST and MASCOT searches in each experiment. Briefly, putative interactors had to be present in all 3 experiments with at

least 4 unique peptides in 2 of these and had to be absent from control pull downs. We excluded all proteins with exclusive nuclear and membrane localisation.

**DT40 constructs**	
HSET KO LA F (Kpn1)	ccaggtaccGATGAGATCTTCCTCTCATCA
HSET KO LAR(Bamh1)	ccactcgagTCGCCGCAATCGCTTCCTCACT
HSET KO RA F (Bamh1)	ccaggatccTTCCTGGAGATCTACAACGAGTCG
HSET KO RA R (Not1)	ccagcggccgc GCAGATCTCAGCCTGAGGTGC
Integration check LA F	AGGGACTGCACATGGGATGA
HSET KO absence F	ATGGCGGCGGTGGGGTCTGGAGGGA
HSET KO R absence R	AGGCCCTGCTCGGGAGCCGC
CEP215 ΔN LA F (Apa1)	ccagggcccTTATAGTCCAGTAATCTCGCCA
CEP215 ΔN LA R (Bamh1)	cgcggatccCTAAATTAAAAGAAGAACATAG
CEP215 ΔN RA F (Bamh1)	cgcggatccAGTTTGGATCATTAATTCCT
CEP215 ΔN RA R (Not11)	ccagcggccgcATTTCAACCAGACTGGACACT
Integration check LA F	ATATTAGCAGCATTTCATCTTAG
CEP215ΔN absence F	GGTGCAGACCTTTAAGGAATCA
CEP215ΔN absence R	AAAAGTAGTGTTCCTCAGAATG
CEP215 ΔHBR LA F (Kpn1)	ccaggtaccACCTCTATTGCCATGCAGAT
CEP215 ΔHBR LA F (Bamh1)	cgcggatccCTAACAAAGGAAAACAATGAT
CEP215 ΔHBR RA F (Spe1)	cgcactagtGTGAGTTTCATATAACTGTGA
CEP215 ΔHBR RA R (Not1)	ccagcggccgcTGTGTTGTTTGCAGAAACAT
Integration check LA F	AGTAAATGGTAGCTGTTTGCTT
CEP215 ΔHBR absence F	AAATCTGATGAAACAATTA
CEP215 ΔHBR absence R	CTCTAACTCTCTGACTATCCTG
**Human constructs**	
CEP215 ΔHBR F	ACAGAGCTTCTGGCTAGC
CEP215 ΔHBR R	ATCTTTTTCATTGAATTTCTGAAGC
CEP215 1F	ggggacaagtttgtacaaaaaagcaggcttcATGATGGACTTGGTGTTG
CEP215 aa300 F	ggggacaagtttgtacaaaaaagcaggcttcGAAGAGGACCTGAGAGAG
CEP215 aa500 F	ggggacaagtttgtacaaaaaagcaggcttcGATTTGGAAGTAATACAG
CEP215 aa700 F	ggggacaagtttgtacaaaaaagcaggcttcACAGAACAAACAGAGCTT
CEP215 aa900 F	ggggacaagtttgtacaaaaaagcaggcttcCCGATCAACACTGCACTC
CEP215 aa 300R	ggggaccactttgtacaagaaagctgggtcctaCTTCTCCTGGACTCTCTC
CEP215 aa600 R	ggggaccactttgtacaagaaagctgggtcctaCTCGTCCTACACGAAAGT
CEP215 aa700 R	ggggaccactttgtacaagaaagctgggtcctaTCTCTTGTTTGTCTCGAA
CEP215 aa 900 R	ggggaccactttgtacaagaaagctgggtcctaGGCTAGTTGTGACGTGAG
CEP215 aa1190 R	ggggaccactttgtacaagaaagctgggtcctaCTTTTGAAGCTGAAATAT
HSET aa1 F	ggggacaagtttgtacaaaaaagcaggcttcaTGGCGGCGGTGGGGTCTGGA

HSET aa673 R	ggggaccactttgtacaagaaagctgggtcctaTGACTTCCGGTTGGCGTG
HSET aa150 R	ggggaccactttgtacaagaaagctgggtcctaGACCTAAATGCAGAACTA
HSET aa151 F	ggggacaagtttgtacaaaaaagcaggcttcAAACGGTGCCGTGAGAGG
HSET aa300 R	ggggaccactttgtacaagaaagctgggtcctaGAGTTCCTGCAGCTGGTT
HSET aa300 F	ggggacaagtttgtacaaaaaagcaggcttcaACCAGCTGCAGGAACTCAA
HSET aa 673 R	ggggaccactttgtacaagaaagctgggtcctaTCACTTCCTGTTGGCCTGAGC

Supplementary Table 2. List of primers used in the study.
